# Genome sequencing of two *Neorhizobium galegae* strains reveals a *noeT* gene responsible for the unusual acetylation of the nodulation factors

**DOI:** 10.1186/1471-2164-15-500

**Published:** 2014-06-19

**Authors:** Janina Österman, Joanne Marsh, Pia K Laine, Zhen Zeng, Edward Alatalo, John T Sullivan, J Peter W Young, Jane Thomas-Oates, Lars Paulin, Kristina Lindström

**Affiliations:** Department of Food and Environmental Sciences, University of Helsinki, Viikinkaari 9, 00790 Helsinki, Finland; Institute of Biotechnology, University of Helsinki, Viikinkaari 9, 00790 Helsinki, Finland; Department of Environmental Sciences, University of Helsinki, Viikinkaari 2a, 00790 Helsinki, Finland; Department of Microbiology and Immunology, University of Otago, Dunedin, 9054 New Zealand; Department of Biology, University of York, Heslington, York YO10 5DD UK; Department of Chemistry, University of York, Heslington, York YO10 5DD UK; Centre of Excellence in Mass Spectrometry, University of York, Heslington, York YO10 5DD UK

**Keywords:** *Neorhizobium galegae*, Genome, Nod factor, *noeT*, Orthologs, Mass spectrometry, Conjugative plasmid

## Abstract

**Background:**

The species *Neorhizobium galegae* comprises two symbiovars that induce nodules on *Galega* plants. Strains of both symbiovars, orientalis and officinalis, induce nodules on the same plant species, but fix nitrogen only in their own host species. The mechanism behind this strict host specificity is not yet known. In this study, genome sequences of representatives of the two symbiovars were produced, providing new material for studying properties of *N. galegae*, with a special interest in genomic differences that may play a role in host specificity.

**Results:**

The genome sequences confirmed that the two representative strains are much alike at a whole-genome level. Analysis of orthologous genes showed that *N. galegae* has a higher number of orthologs shared with *Rhizobium* than with *Agrobacterium*. The symbiosis plasmid of strain HAMBI 1141 was shown to transfer by conjugation under optimal conditions. In addition, both sequenced strains have an acetyltransferase gene which was shown to modify the Nod factor on the residue adjacent to the non-reducing-terminal residue. The working hypothesis that this gene is of major importance in directing host specificity of *N. galegae* could not, however, be confirmed.

**Conclusions:**

Strains of *N. galegae* have many genes differentiating them from strains of *Agrobacterium*, *Rhizobium* and *Sinorhizobium*. However, the mechanism behind their ecological difference is not evident. Although the final determinant for the strict host specificity of *N. galegae* remains to be identified, the gene responsible for the species-specific acetylation of the Nod factors was identified in this study. We propose the name *noeT* for this gene to reflect its role in symbiosis.

**Electronic supplementary material:**

The online version of this article (doi:10.1186/1471-2164-15-500) contains supplementary material, which is available to authorized users.

## Background

Genome sequencing has become an important tool for studying microbial properties, shedding light on phenotypes specific to different strains and environments as well as on evolutionary patterns. Genome sequences give researchers the opportunity to study genetic traits in a broader context, providing more information and the possibility to detect new linkages. The α-proteobacterial species *Neorhizobium galegae* is a plant root-nodulating nitrogen-fixing bacterium which is interesting in several ways. It was described by Lindström in 1989 [1] as *Rhizobium galegae* and renamed by Mousavi *et al*. in 2014 [2]. Phylogenetically, *N. galegae* differs from many of the well-known *Rhizobium* species, being more closely related to *Agrobacterium* than are many other nitrogen-fixing bacteria (e.g. [3, 4]). The best-studied strains of *N. galegae* are those nodulating plants in the genus *Galega*: *G. orientalis* Lam. and *G. officinalis* L. These strains are very host specific, forming effective nodules only on the aforementioned *Galega* species. The former species *Rhizobium galegae* included only *Galega*-nodulating strains, whereas the new species *N. galegae* also includes strains infecting plant species from a range of legume genera, including *Astragalus*, *Caragana*, *Lotus*, *Medicago*, *Sesbania* and *Vigna* [2]. In this paper, however, we will consider only the *Galega*-nodulating strains. No other rhizobial species is known to induce root nodules on *Galega* legumes. The factors determining this strict host specificity of *N. galegae* have not been revealed by the information currently available, which is why a more complete set of information is needed. During the last ten years, the genomes of many strains phylogenetically related to *N. galegae* have been sequenced. This increasing amount of genomic information brings new possibilities to study not only the evolutionary relationship between these species, but also inter- and intraspecific genomic differences that can contribute to the establishment of the symbiosis and influence survival in the soil environment. The symbiotic behaviour of the *Galega*-nodulating strains of *N. galegae* makes this species an attractive candidate for this kind of study. These *N. galegae* strains are divided into two symbiovars (sv.) according to which one of the two host plant species they nodulate effectively [5]. Strains of both symbiovars are able to induce nodules on both *G. orientalis* and *G. officinalis*, but effective nodules are only formed on *G. orientalis* by sv. orientalis strains, and on *G. officinalis* only by sv. officinalis strains. None of the *N. galegae* strains tested so far induce nitrogen-fixing nodules on both *G. orientalis* and *G. officinalis*. The mechanism behind this strict discrimination is still unknown. However, while both symbiovars were known to induce root nodules only on *Galega* species, nodule formation has recently been observed on some species of *Acacia* (our unpublished data).

Rhizobia produce signal molecules called Nod(ulation) factors (NFs) upon induction of the *nod* genes. These genes are activated as a response to external signals, usually in the form of flavonoids exuded from plant roots but occasionally also by environmental factors such as high salt concentration [6]. NFs are lipochitin oligosaccharides (LCOs), consisting of a backbone of mainly three to five β-1,4-linked N-acetylglucosamine (GlcNAc) residues, with an N-acyl group substituted on the non-reducing-terminal monosaccharide residue. Individual rhizobial species produce NFs with different chemical substituents on the GlcNAc residues, with most of these substituents being found on the reducing- and non-reducing-terminal residues. These signalling molecules are important for the establishment of a well-functioning symbiosis with legume hosts, and variations in the structure of the LCOs are known to be important for host specificity. NFs of rhizobia have been widely studied (for reviews see, for example, [7]– [10]) but the exact mechanisms by which the different structures are perceived by the host and the host signalling pathways they elicit, are the subject of ongoing studies. The NFs of *N. galegae* carry an acetyl substituent on the GlcNAc residue adjacent to the non-reducing-terminal residue, which is an unusual, but not unique, location for substitution among the many NF structures described. This decoration was first described in 1999 [11], and it has since been hypothesized that this decoration is an important factor contributing to the strict host specificity of this species. However, to date, the gene responsible for adding the acetyl group to the LCOs has not been identified.

In order to gain access to more information that can be used to unravel the mechanism(s) that contribute to *N. galegae* host specificity, we sequenced the complete genomes of one representative each of the two symbiovars of *N. galegae*: the type strain HAMBI 540^T^ [EMBL:HG938353-HG938354], representing sv. orientalis, and strain HAMBI 1141 [EMBL:HG938355-HG938357], a representative strain of sv. officinalis. In the present study, the *N. galegae* genome sequences were compared to each other, to complete genomes of other rhizobial species and a representative of the genus *Agrobacterium*. The aim was to determine the degree of divergence between the symbiovars and the overall genetic similarity of *N. galegae* to closely related species, and to reveal and investigate differences that might play a role in nodulation specificity. Analysis of the genomic region containing the symbiotic *nod*, *nif* and *fix* genes of *N. galegae* revealed a previously unknown gene, potentially responsible for *O*-acetylation of the Nod factor. In order to demonstrate the function of this gene, which we call *noeT*, a deletion mutant was constructed. The structures of NFs produced by this mutant strain and its wild type parental strain were studied by mass spectrometry, and plant inoculation tests were performed to study the impact of the mutation on nodulation and nitrogen fixation. The genome sequences also revealed two sets of genes involved in conjugational transfer on the replicons of strain HAMBI 1141. Experiments were performed to find out if the plasmid containing the symbiosis genes is conjugative.

## Results

### General features of the genomes of HAMBI 540^T^ and HAMBI 1141

**Figure 1:**
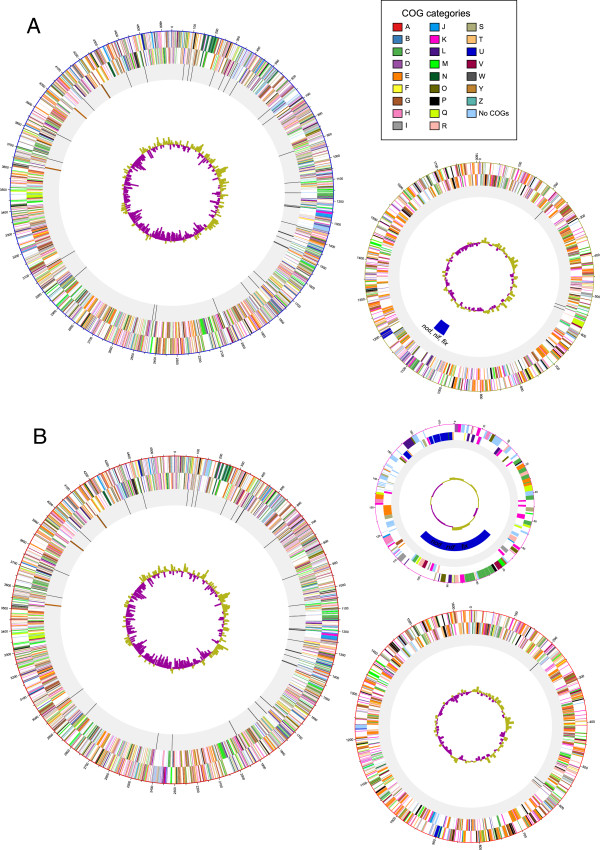
**Circular representation of the sequenced genomes. A)** *N. galegae* sv. orientalis HAMBI 540^T^ **B)** *N. galegae* sv. officinalis HAMBI 1141. The circles represent, from outer to inner, CDSs on the forward strand, reverse strand, rRNA (purple) and tRNA (orange) genes on grey background, sym genes marked with blue on the replicons that contain these, and GC-skew. The CDSs are coloured according to the COG category they are assigned to (colour key in upper right corner of the figure).

Essential information on the genomes of *N. galegae* strains HAMBI 540^T^ and HAMBI 1141 is presented in Table 1. In addition to the chromosomes, both strains harbour large megaplasmids (HAMBI 540^T^ 1.81 Mb, HAMBI 1141 1.64 Mb) which have (i) plasmid-type *repABC* replication systems, (ii) a G + C composition within 1% of the host’s chromosome and (iii) orthologues of chromosomally located core genes in other species, even when the search criteria included a cut-off threshold as high as 70% amino acid identity over practically the whole protein sequence. Thus, the features of these megaplasmids fulfil the chromid criteria [12] and, hence, hereafter will be called chromids. The HAMBI 540^T^ genome consists of only two replicons, while HAMBI 1141 possesses a third replicon which is a 175 kb plasmid. Surprisingly, the symbiosis genes of strain HAMBI 1141 are located on this small plasmid, not on the chromid. Codon usage analysis showed that both *N. galegae* strains use all 64 codons, even though only 50 and 51 tRNAs were found respectively. The rate of usage of any single codon in one strain was proportional to that of the other strain. Thus, at a general level, codon usage is not remarkably different between the two genomes. Figure 1 shows the genomes as circular representations of each replicon.

**Table 1:** **The genomes of** ***N. galegae*** **strains HAMBI 540** ^**T**^ **and HAMBI 1141 in numbers**

	HAMBI 540 ^T^		HAMBI 1141	
**Total size**		6.45 Mb		6.41 Mb
**Replicons**	chromosome	4.65 Mb	chromosome	4.60 Mb
	chromid	1.81 Mb	chromid	1.64 Mb
			plasmid	175 kb
**G + C content (%)**	chromosome	61.5	chromosome	61.6
	chromid	60.6	chromid	60.7
			plasmid	57.5
**Total no. of genes**	6230		6213	
**rRNA operons**	3		3	
**tRNAs**	51		50

### Genomic variability among strains of *Neorhizobium*, *Rhizobium*, *Sinorhizobium*and *Agrobacterium*

**Figure 2:**
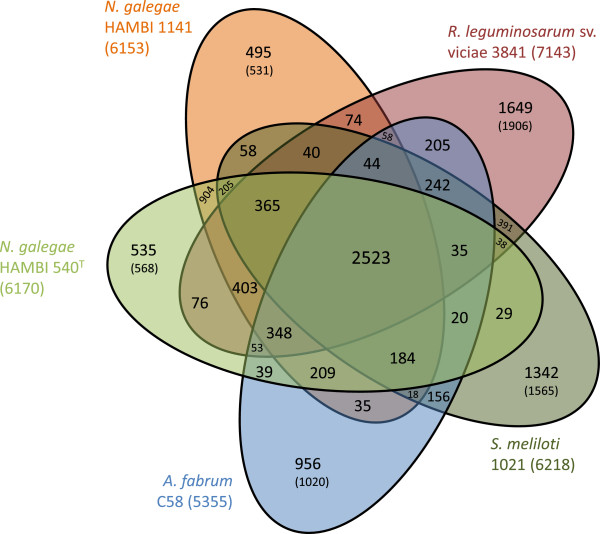
**Illustration of the results from the OrthoMCL analysis with five genomes.** Each strain has its own colour and an indication of the total number of protein-coding genes indicated in parenthesis next to the strain name. The number in the middle is the number of ortholog groups shared by all five strains. The number of singletons (i.e. genes for which no orthologous gene was found) is indicated for each strain, with the number in parenthesis indicating the total number of strain-specific genes (singletons defined by OrthoMCL together with the genes from ortholog groups consisting of multiple genes from one strain only).

The two *N. galegae* genomes were compared to three related species at the protein level through analysis of orthologous genes. The strains used in this analysis were model strains of the genera *Rhizobium*, *Sinorhizobium* (syn. *Ensifer*) and *Agrobacterium*. There were 2523 ortholog groups shared by all five strains. These groups contained 2608 genes of *N. galegae* HAMBI 540^T^ and HAMBI 1141 each, 2614 genes of *R. leguminosarum* sv. viciae 3841, 2693 genes of *S. meliloti* 1021 and 2651 genes of *A. fabrum* C58. Analysis of inter-specific ortholog groups in relation to reference strain proteome size showed that *N. galegae* shares the most orthologs with *R. leguminosarum* sv. viciae 3841 (403 ortholog groups shared by the three strains, containing 425 genes of strain 3841, i.e. 5.9% of its proteome size, Figure 2). The smallest number of inter-specific ortholog groups was found with *S. meliloti* 1021 (3.5%). Moreover, there were 365 ortholog groups where all strains but *A. fabrum* C58 were represented. Compared to 209 ortholog groups shared by the two *N. galegae* strains and *A. fabrum* C58, this analysis indicates that *N. galegae* has more in common with the strains representing the rhizobial species examined than it has with *A. fabrum* C58. The analysis also showed that *S. meliloti* 1021 seems more closely related to *N. galegae* HAMBI 1141 than to HAMBI 540^T^. *S. meliloti* 1021 has twice as many pairwise ortholog groups shared with HAMBI 1141 compared to the number of orthologs shared with HAMBI 540^T^ (58 compared to 29, Figure 2). In contrast, a proportional number of ortholog groups are shared when *R. leguminosarum* sv. viciae 3841 or *A. fabrum* C58 is compared to *N. galegae* HAMBI 1141 and HAMBI 540^T^.

Those genes identified as singletons (i.e. genes not assigned to any ortholog group) in the *N. galegae* strains after OrthoMCL analysis, were distributed over the whole genome (see Additional file 1: Figure S2). A majority of singletons identified were genes not assigned to any COG category. In both strains, genes involved in amino acid transport and metabolism were much more abundant among singletons on the chromids than on the chromosomes. In addition, singletons involved in transcription and inorganic ion transport and metabolism were overrepresented on the chromid of HAMBI 540^T^ compared to singletons on its chromosome. Analysis of the genomic location of genes from *N. galegae*-specific ortholog groups (i.e. the 904 groups present in both *N. galegae* strains but not the other species, Figure 2), showed that these genes had largely syntenic locations in the two strains (see Additional file 1: Figure S3).

**Figure 3:**
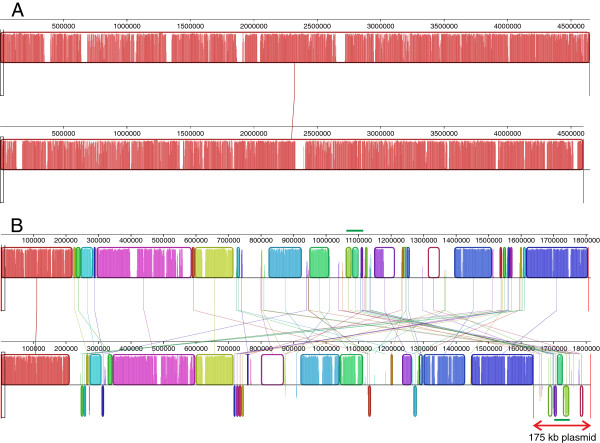
**Alignments of the** ***N. galegae*** **genome sequences. A)** Comparison of the chromosomes of strains HAMBI 540^T^ (top) and HAMBI 1141 (bottom). **B)** Comparison of the HAMBI 540^T^ chromid (top) with the 1141 chromid having the 175 kb plasmid sequence concatenated at the end of the chromid sequence (bottom). The symbiosis genes are indicated with green horizontal bars.

The genome nucleotide sequences of *N. galegae* HAMBI 1141 and HAMBI 540^T^ were aligned to provide an overall picture of the synteny between the two genomes (Figure 3). The chromosome sequences were highly syntenic and only relatively short chromosomal regions unique to each strain were apparent. The chromids also have a degree of shared synteny, but regions where genetic rearrangements have occurred and regions lacking obvious homology comprise a considerable proportion of these replicons. Aside from the symbiosis genes, there is not much homology found between the 175 kb plasmid of HAMBI 1141 and the chromid of HAMBI 540^T^.

**Figure 4:**
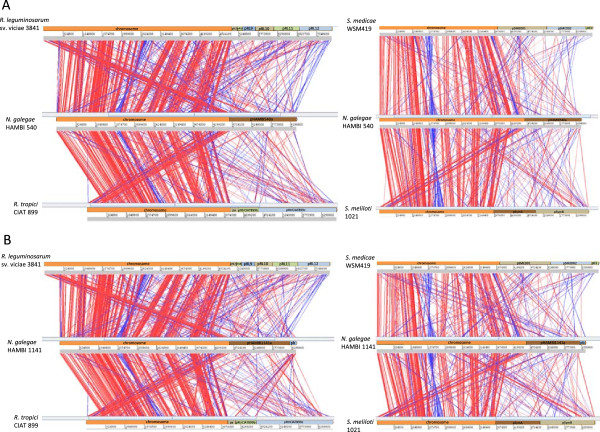
**Genomic alignments of** ***N. galegae*** **compared to other rhizobial genomes. A)** Left: genomic alignment of *R. leg*uminosarum sv. viciae 3841 (top), *N. galegae* HAMBI 540^T^ (middle) and *R. tropici* CIAT 899 (bottom). Right: genomic alignment of *S. medicae* WSM419 (top), *N. galegae* HAMBI 540^T^ (middle) and *S. meliloti* 1021 (bottom). **B)** Left: genomic alignment of *R. leg*uminosarum sv. viciae 3841 (top), *N. galegae* HAMBI 1141 (middle) and *R. tropici* CIAT 899 (bottom). Right: genomic alignment of *S. medicae* WSM419 (top), *N. galegae* HAMBI 1141 (middle) and *S. meliloti* 1021 (bottom). Red connecting lines indicate syntenic regions, blue connecting lines indicate inverted regions.

In order to further analyse the structural variability between the two *N. galegae* genomes and related rhizobial strains, alignments to complete genomes of *R. leguminosarum*, *R. tropici*, *S. medicae* and *S. meliloti* were generated (Figure 4). This analysis confirmed that, among these strains, *N. galegae* is most closely related to *R. leguminosarum*. Generally, chromosomes among these strains have a fairly high shared synteny, while the *N. galegae* chromids (pHAMBI540a and pHAMBI1141a) contain genetic fragments dispersed throughout the reference genomes at a much higher frequency. A majority of these chromids do, however, consist of genetic regions with no detectable similarity to the reference genomes. The plasmid pHAMBI1141b has a very limited number of regions with similarity to regions on the reference genomes. There is clearly more similarity with regions on the two *Rhizobium* genomes (11 and 21 matches to *R. leguminosarum* and *R. tropici* respectively) than there is with the *Sinorhizobium* genomes (3 matches to *S. medicae*, none to *S. meliloti*). However, among the 21 matches to the *R. tropici* genome, 13 matches correspond to a single region on pHAMBI1141b; a probable transposase gene region. The total length of the pHAMBI1141b regions having a match on the *R. tropici* genome is only 24 kb (out of the whole 175 kb plasmid). Most parts of the plasmid did not show homology to other rhizobial or agrobacterial plasmids when aligned to plasmids of 11 rhizobial strains from the genera *Rhizobium*, *Sinorhizobium* and *Mesorhizobium* (*R. leguminosarum* sv. viciae 3841, *R. leguminosarum* sv. trifolii WSM2304, *R. tropici* CIAT 899, *R. phaseoli* CIAT 652, *R. etli* sv. mimosae str. Mim1, *R. etli* CFN 42, *S. fredii* NGR234, *S. meliloti* 1021, *S. medicae* WSM419, *M. ciceri* sv. biserrulae WSM1271, *R. rhizogenes* K84) and *A. fabrum* C58. When megablast was used to search for similarities of the pHAMBI1141b plasmid to sequences in the NCBI nr database, the replicon generating the highest similarity (in terms of summed up alignment lengths) was pRtrCIAT899b of *R. tropici* CIAT 899 (a total of 25 kb matching sequence).

The RepABC proteins of the same strains that were used for alignment with the plasmid pHAMBI1141b were also used for analysis of the evolution of the chromids and plasmid in the two *N. galegae* strains. The evolutionary history was inferred by a Maximum Likelihood phylogeny (Additional file 1: Figure S1). The analysis showed that the replication systems of the two *N. galegae* chromids are very similar, but not very closely related to any of the other replicons analysed. The replication system of the plasmid pHAMBI1141b is different from the corresponding system on the chromid, closely related to especially plasmid pRtrCIAT899c of *R. tropici* CIAT 899, but also to plasmid pC58At of *A. fabrum* C58. However, the relatedness of the replication systems does not reflect the overall similarity between these plasmids.

### Genomic features related to the ecology of *N. galegae*

Genes that are interesting with regard to ecological interactions of rhizobia include genes related to polysaccharide production, denitrification, pilus formation and conjugation. Rhizobial surface polysaccharides have been proven important for symbiosis-related functions. In *N. galegae*, genes for exopolysaccharide production, namely succinoglycan (EPS I) production, similar to those found in *S. meliloti* [13], are present (RG1141_CH33450-CH33590, RG540_CH34270-CH34410). However, in contrast to the gene organisation in *S.meliloti*, the *exoT*, *exoI*, and *exoZ* genes were not found in *N. galegae*. An *exoB* gene was found distantly from the other *exo* genes (RG1141_CH28070, RG540_CH28720). A possible *exoR* regulator gene was also detected (RG1141_CH14030, RG540_CH14810). A gene cluster homologous to the *acpXL*-*lpxM*(*msbB*) gene cluster of *S. meliloti* [14], responsible for biosynthesis and incorporation of the acyl chain substituent of lipid A in the lipopolysaccharide (LPS), is also present in *N. galegae* (RG1141_CH19440-CH19390, RG540_CH20240-CH20190), as well as other genes involved in lipid A biosynthesis, namely a *bamA*-*lpxD*-*fabZ*-*lpxA*-*lpxI*-*lpxB* gene cluster (RG1141_CH15400- CH15450, RG540_CH15850-CH15900) and an *lpxK*-like gene (RG1141_CH04800, RG540_CH04440). Genes involved in LPS core oligosaccharide synthesis are also present on the chromosome: *greA-lpsB* (RG1141_CH24690-CH24700, RG540_CH24720- CH24750), *lpsE* (RG1141_CH24710, RG540_CH24760), a glycosyl transferase (RG1141_CH24720, RG540_CH24770) and an *lrp* gene (RG1141_CH24730, RG540_CH24780). Genes responsible for capsular polysaccharide (KPS) synthesis, export and polymerization in *N. galegae* could not be pinpointed based on sequence homology to known rhizobial *rkp-1*, *rkp-2* and *rkp-3* region genes. Even though strains of *N. galegae* have been shown to produce LPS containing different O-antigen chains [15], no genes for O-antigen transport (*wzm* and *wzt*) could be detected in either *N. galegae* strain.

In addition to polysaccharide production, interaction between bacteria and plants can be enhanced by extracellular structures like the Flp/Tad pilus, which has been proposed to play a possible role in virulence of the potato pathogen *Pectobacterium* [16]. Annotation revealed some *tad* genes (RG1141_CH43400, RG1141_CH43410, RG1141_CH43500, RG1141_CH43510, RG540_CH43790, RG540_CH43800, RG540_CH43890, RG540_CH43900, RG540_CH10650, RG540_CH10660) on the chromosome of *N. galegae*, even though no two-component system like that one found in *Pectobacterium* could be detected in the vicinity of these genes. In addition, the similarity of the *tad* genes of *N. galegae* to those of *Pectobacterium* is very limited. Nonetheless, based on blastx results, gene regions similar to that in *N. galegae* seem to be common in rhizobial relatives.

Even though rhizobia are known for their ability to fix nitrogen, some strains have genes encoding functions in the denitrification pathway. The only rhizobia shown to be true denitrifiers belong to the genus *Bradyrhizobium*, but partial denitrification pathways have also been found in species belonging to other genera [17]– [19]. Denitrification functions are encoded by the *nap*, *nir*, *nor* and *nos* gene clusters. In *N. galegae* strains HAMBI 540^T^ and HAMBI 1141, the *nirKV* (RG1141_CH34420-CH34410, RG540_CH35240-CH35230) and *norECBQD* (RG1141_CH34790-CH34840, RG540_CH35550-CH35600) genes (as well as a putative *norF* between *norE* and *norC*) are present, encoding nitrite and nitric oxide reductase respectively. However, no genes for nitrate reductase or nitrous oxide reductase have been detected.

An important part of the genomic differences between the sequenced strains is made up of two gene regions encoding putative type IV secretion systems (T4SS). Genes coding for a type IVB rhizobial plasmid conjugation system [20] are located on the chromid of strain HAMBI 1141 (Mfp component RG1141_PA08510-PA08620, Dtr component RG1141_PA08710-PA08730) while a type I conjugation system (Mfp component and QS regulation genes RG1141_PB01500-PB01560, Dtr component RG1141_PB01600-PB01730) is found on the symbiosis plasmid. The *traI*/*traR*/*traM* quorum sensing regulation system is present on the plasmid pHAMBI1141b together with what seems to be a complete set of genes required for a functional T4SS system. Experimental work showed that strain HAMBI 1141 is able to transfer its symbiosis plasmid through conjugation. Plant tests and plasmid profile investigation by a modified Eckhardt gel procedure confirmed that conjug transfer of the plasmid carrying the symbiosis gene region in strain HAMBI 1207 (streptomycin-resistant derivative of HAMBI 1141) occurred when the nodulation-defective strain HAMBI 1587 (*N. galegae* sv. orientalis), was mated with strain HAMBI 1207. These transconjugants formed effective nodules on *G. officinalis*, indicating that all genes needed for nitrogen fixation on *G. officinalis* were transferred to strain HAMBI 1587. The other genes present in strain HAMBI 1587 did not interfer with symbiosis on *G. officinalis*. On the other hand, the same transconjugants formed effective nodules on *G. orientalis* only sporadically. This observation indicates that even though the nodulation defect was complemented by the *nod* genes on the conjugated plasmid, some of the genes present on this same plasmid interfere with the symbiotic functions on *G. orientalis*.

To further investigate whether the T4SS genes on the chromid of the same strain were involved in transfer of the symbiosis plasmid, a deletion mutant of HAMBI 1141 lacking the defined putative T4SS genes on the chromid (HAMBI 3490), was constructed using a Cre-*loxP*-based technique. Two *lox* sites were inserted flanking the target region, which was then excised from the chromid by the Cre protein introduced on a separate vector. When conjugation was attempted between HAMBI 3490 and HAMBI 1587, no nodules could be observed on the inoculated *G. orientalis* plants, indicating that the chromid-borne T4SS genes are required to mobilise the symbiosis plasmid. To further investigate this hypothesis, a plasmid-cured derivative of strain HAMBI 1207 (assigned HAMBI 3489) was constructed using random transposon mutagenesis of the suicide vector pMH1701 containing the *sacB* gene. HAMBI 3489 was conjugated with HAMBI 3490 and the wild-type HAMBI 1141 separately. Exconjugants were inoculated on *G. officinalis* plants to determine whether conjugal transfer of the symbiosis plasmid had taken place, thereby rendering HAMBI 3489 able to induce nodules on *G. officinalis*. However, no nodules could be observed on any of the inoculated plants, regardless of whether HAMBI 3489 had been mated with the wild-type or the deletion mutant of HAMBI 1141. To ensure that the results were not due to unsuccessful selection of transconjugants when streptomycin was used to select for the recipient, additional conjugation attempts were performed with a derivative of HAMBI 3489 containing a gene for gentamicin resistance (HAMBI 3491) as the recipient. HAMBI 3491 was mated with HAMBI 3490, HAMBI 3470 (HAMBI 1587 which has gained the symbiosis plasmid of HAMBI 1207) and HAMBI 1141. Plasmid profile analysis and re-inoculation tests indicated that there were no true transconjugants resulting from these matings. Taken together, all of these results indicate that the symbiosis plasmid in HAMBI 1141 is not self-transmissible, but likely needs some assistance from the T4SS genes on the chromid for transfer. In addition, conjugal transfer of the symbiosis plasmid in HAMBI 1141 does not seem to occur at a high frequency to a broad range of recpipients, since no true transconjugants could be observed when HAMBI 1141 was mated with *A. fabrum* strain C58C1 (cured of its Ti plasmid), *S. meliloti* HAMBI 1213 (NodC^-^) and *R. leguminosarum* sv. viciae HAMBI 1594 (NodA^-^) and exconjugants tested on *G. officinalis* as well as the hosts *Medicago sativa* (*A. fabrum* and *S. meliloti*) and *Vicia villosa* (*R. leguminosarum*).

In strain HAMBI 540^T^, no T4SS-related genes are found. On the other hand, a type VI secretion system (T6SS) is found on the chromid of strain HAMBI 540^T^ (RG540_PA11400-PA11590), while no corresponding secretion system can be found in strain HAMBI 1141. This T6SS comprises the 14 genes found in the *imp* operon of *A. fabrum* strain C58 as well as the three conserved genes of the *hcp* operon; *tssH*, *tssD* and *tssI* [21]. The T6SS of HAMBI 540^T^ is most similar to systems found in three other rhizobial strains: *R. etli* sv. mimosae strain Mim1 plasmid pRetMIM1f (NC_021911), *R. leguminosarum* sv. viciae strain 3841 plasmid pRL12 (NC_008378.1), and *Rhizobium* sp. BR816 scaffold 1_C5 (AQZQ01000005.1). Possible imperfect σ^54^ and NifA binding sites were found in the upstream region of *tssA*, indicating that the T6SS might play a role in symbiosis.

### Symbiosis gene regions of *N. galegae*

**Figure 5:**
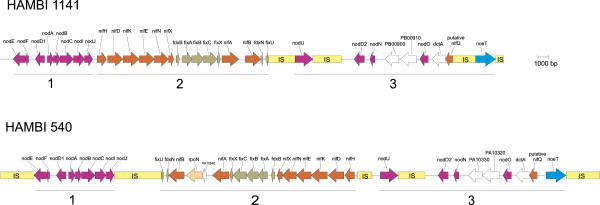
**Schematic representation of the symbiotic gene regions of the strains HAMBI 1141 and HAMBI 540** ^**T**^ **.** The three main blocks of genes are marked with numbers 1–3. The regions are shown in scale.

As can be seen in Figure 3, there are genetic rearrangements found inside the regions comprising the symbiosis genes of the two strains studied. Insertion sequences (ISs) are well represented in these regions, probably accounting for some of the rearrangements. Nevertheless, the known symbiosis genes form three blocks that are represented in the same configuration in both genomes (Figure 5). In strain HAMBI 540^T^, these blocks are flanked by IS elements, while strain HAMBI 1141 has an IS at the right boundary only, downstream of *noeT*. In this strain, *nodE* is separated from the next transposase gene by 38 genes, some of which are also encountered in the region downstream of *nodE* in strain HAMBI 540^T^. The regions downstream of the *noeT* gene are, however, different in the two strains. The borders of the symbiosis gene cluster are not defined, but here we concentrate on the regions that contain the known symbiosis genes. The genomic blocks assigned numbers 2 and 3 in Figure 5 are not entirely identical in the two strains. In block 2, an *rpoN* gene (RNA polymerase σ^54^) has been inserted between *nifA* and *nifB* in HAMBI 540^T^. In block 3, HAMBI 1141 harbours an IS upstream of *noeT*, while there is no IS in this part of the region in HAMBI 540^T^. It is worth noting that the IS elements present in the symbiosis gene regions of the two strains in this study are not highly similar. Despite the impression of similarity of the ISs surrounding the *nodU* gene, these regions seem unrelated.

Three genes present in the symbiosis gene regions did not at first glance seem to be involved in symbiotic events. The genes PA10320 and PA10330 of HAMBI 540^T^ (and corresponding genes PB00910 and PB00900 in HAMBI 1141) appear to form a kind of type I secretion system (T1SS). Based on nucleotide sequence similarity the two genes were most similar to the rhizobiocin secretion system *rspDE*-like genes of *R. tropici* CIAT 899 [22] and *R. leguminosarum* [23], when the region containing the two genes was compared to genomic data of other rhizobia. However, the product of gene PA10320 was most similar to a PrtD family type I secretion system ATP binding cassette (ABC) gene product, while the product of PA10330 was most similar to a HlyD family type I secretion system membrane fusion protein gene product. The product of the third unexpected gene, a second copy of *dctA*, is a C4-dicarboxylic acid transporter protein. The putative *nifQ* gene upstream of it has similarity to the *nifQ* gene of other species, although the product has regions with very little similarity to other NifQ protein sequences. More importantly, the proposed molybdenum-binding motif Cx_4_Cx_2_Cx_5_C [24] is not present in this protein in the two *N. galegae* strains.

**Figure 6:**
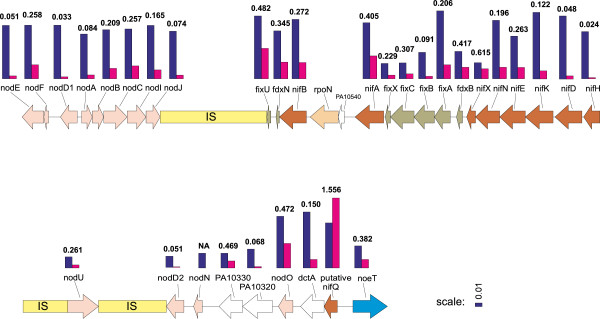
**Illustration of** ***d***_***N***_ **/** ***d***_***S***_ **of the symbiosis genes of the two** ***N. galegae*** **strains.** The HAMBI 540^T^ symbiosis gene region used as model sequence. The blue bars represent the rate of synonymous substitutions *d*_*S*_, and the magenta-coloured bars the rate of nonsynonymous substitutions *d*_*N*_. The numbers on top of the bars are the *d*_*N*_/*d*_*S*_ ratios.

Analysis of nonsynonymous/synonymous substitution rate ratio was performed for the genes in the symbiosis gene region (Figure 6) in a pairwise manner, comparing the genes of the two sequenced strains. Averaging over the whole gene, a *d*_N_/*d*_S_ < 1 was obtained for most genes, indicating fairly strong purifying selection (Figure 6). However, the putative *nifQ* gene had a non-synonymous substitution rate that was much higher than for any other gene, and much higher than the synonymous substitution rate of the same gene, making this gene a factor of interest. An analysis of variable selective pressure acting on different branches of the NifQ phylogeny of rhizobial species (Additional file 1: Figure S4) was performed to investigate whether *N. galegae nifQ* has changed under positive selection. The estimate of *ω* under the null hypothesis, as an average over the phylogeny, was 0.25, indicating that evolution of *nifQ* was dominated by purifying selection. The likelihood ratio test (LRT) suggests that selective pressure (0.26) on the branch separating the *N. galegae* symbiovars from their closest neighbour (*S. fredii*) in the ML tree, was not significantly different from the average over the other branches. Hence, there is no evidence for functional divergence of *nifQ* of *N. galegae* compared to the others, by positive selection. When testing the hypothesis that the divergence of *N. galegae nifQ* was due to an increase in the non-synonymous substitution rate over all lineages of *N. galegae*, the LRT was highly significant (P < 0.0001), and the parameter estimates for *N. galegae nifQ* indicated an increase in the relative rate of non-synonymous substitution by a factor of 3. These results indicate that the non-synonymous rate increased in *N. galegae nifQ* following divergence from other rhizobial species, even though it seems to have occurred through an increase in the mutation rate rather than by positive selection. The function of *nifQ* in *N. galegae* has not been investigated to date.

### *noeT*is involved in Nod factor biosynthesis

The whole-genome sequence analysis confirmed previous work on the *N. galegae* symbiosis gene region [25, 26], and enabled an extended analysis of this region. As a result, a previously undiscovered putative nodulation gene, preceded by a nod-box sequence, was identified at the rightmost end (according to the arrangement in Figure 5). Homology searches performed using NCBI BLAST suggested that this gene was a putative acetyltransferase gene, the closest homolog (95% and 96% amino acid identity with the HAMBI 1141 and HAMBI 540^T^ proteins respectively) being a gene called *hsnT* (*h*ost *s*pecific *n*odulation gene *T*) in *R. leguminosarum* sv. trifolii ICC105 (accession number EU919402). The Nod factors of *N. galegae* have been shown to have an unusual acetyl substituent on the GlcNAc residue adjacent to the non-reducing-terminal residue, but the gene responsible for this modification had not been determined. Thus, we suspected that this gene could be responsible for adding this decoration and hence, we named this gene *noeT*.

**Figure 7:**
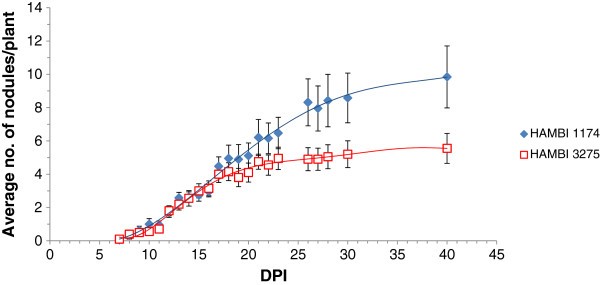
**Average number of nodules observed on** ***G. orientalis*** **plants after inoculation.** Nineteen plants inoculated with wild-type *N. galegae* HAMBI 1174 and twenty plants inoculated with its *noeT* mutant HAMBI 3275 were scored for nodules present between 7 and 40 dpi. Error bars represent standard error.

To investigate whether the *noeT* gene has an impact on symbiosis, a mutant was constructed in strain HAMBI 1174 (Sm/Spc resistant derivative strain of HAMBI 540^T^) background where this gene was replaced by the Ω-Km interposon. When the mutant strain (HAMBI 3275) was inoculated on *G. orientalis*, nodules were formed at the same rate as for the wild-type strain during the first 16 days post-inoculation. After 16 days, new nodules continued to be formed by the wild-type, whereas the number of nodules formed by the mutant increased only slightly (Figure 7). At 40 days post-inoculation, the average number of nodules formed per plant was significantly different between plants inoculated with HAMBI 1174 and HAMBI 3275 at 40 dpi (U = 103.500, z = -2.450, p = 0.014). Because of the growth-limiting test conditions, not all nodules were obviously effective (large and pink). However, the proportion of effective nodules formed (average number of effective nodules compared to total number of nodules) per plant was the same for plants inoculated with either of the strains at 17 dpi (0.6), but differed at 40 dpi (0.7 for HAMBI 1174 and 0.9 for HAMBI 3275). When the mutant strain was tested on *Trifolium repens*, *Pisum sativum* cv. Afghanistan, *Phaseolus vulgaris*, *Vicia hirsuta* and *Astragalus sinicus*, no nodules were observed, as was the case when these plants were inoculated with the wild-type. Ineffective nodules were induced on *G. officinalis*.

The plant tests showed that the mutation did not affect the ability of the bacterium to induce nodules on *Galega* plants nor to fix nitrogen inside the nodules of *G. orientalis*. Nevertheless, the mutation had an impact on the number of nodules formed as time passed. In order to determine the function of the *noeT* gene, cultures of wild type *R galegae* strain HAMBI 1174 and its *noeT* mutant HAMBI 3275 were generated for LCO isolation and structural analysis. The NF extracts were fractionated using solid phase extraction (SPE) with 45% and 60% acetonitrile solutions. The RP-HPLC profiles of the 45% and 60% SPE fractions from the wild type and mutant strain crude Nod factor extracts showed major peaks of UV absorbance for fractions eluting between 40–50 minutes (Additional file 1: Figure S5, regions marked 2 and 3), which have previously been shown to correspond to the elution position of LCOs [27]. In the chromatogram of the 45% SPE fraction, these peaks are dwarfed by a very strongly absorbing peak, on MS analysis shown to be a polymeric contaminant eluting between 30 and 40 minutes (Additional file 1: Figure S5, region marked 1). HPLC fractions from the fractionation of the 45 and 60% SPE fractions of the culture filtrate from wild type and *noeT* mutant *N. galegae* HAMBI 1174 were analysed using ESI-MS and MALDI-MS and collision-induced dissociation (CID) product ion analysis, to determine LCO structures. In fractions eluting between 40–52 minutes, giving rise to strong UV absorbance at 203 nm (Additional file 1: Figure S5), peaks corresponding to [M + H]^+^ and [M + Na]^+^ were observed.

The wild type strain gave LCO-derived [M + H]^+^ peaks at *m/z* 1134 and 1162 along with [M + Na]^+^ peaks at *m/z* 1114, 1118, 1120, 1134, 1136, 1142, 1144, 1146, 1162, 1184, 1186, 1188 and 1190. These ions correspond in composition to GlcNAc_4_-containing LCOs with C18 or C20 fatty acyl chains and substituted with a carbamoyl, acetyl and exceptionally a methyl moiety (Table 2). CID was used to generate product ions that allowed structures to be assigned. Intense product ions were generated from the majority of the LCO-derived signals observed. The *m/z* values of these fragment ions, largely arising by glycosidic bond cleavage and charge retention on the non-reducing-terminal portion (B series ions), allow determination of the fatty acyl chain present on the non-reducing-terminal residue as well as the substituents arranged on the chitin backbone. It is notable that some of the species observed from the wild type strain correspond to LCOs bearing an additional moiety that adds 42 Da, which would correspond to the presence of an acetyl moiety. One of the major LCOs from *N. galegae* HAMBI 1174 resulting in intense mass spectrometric signals ([M + H]^+^ at *m/z* 1162) gave product ions at *m/z* 941, 738 and 493 (Additional file 1: Figure S6) consistent with B-ions for a GlcNAc_4_ species containing a C20:3 fatty acid chain, a carbamoyl group on the non-reducing-terminal residue, and an acetyl moiety present on the GlcNAc unit adjacent to the non-reducing-terminal residue. In addition, fragment ions were observed 60 *m/z* units below the precursor (-60 at *m/z* 1102), the B_3_ ion (-60 at *m/z* 881) and the B_2_ ion (-60 at *m/z* 678), corresponding to the elimination of the acetyl group as neutral acetic acid.

**Table 2:** **Summary of the mass spectrometric data from the LCOs in 45% and 60% SPE fractions**

Parent ion	Molecular species	Fragment ions	Structure assignment
**HAMBI 1174**			
**1114**	[M + Na]^+^	487, 690, 893	IV(C18:3, Cb)
**1118**	[M + Na]^+^	491, 694, 897	IV(C18:1, Cb)
**1120**	[M + Na]^+^	493, 696, 899	IV(C18:0, Cb)
**1134**	[M + H]^+^	465, 710, 913	IV(C18:3, Cb, OAc)
**1134**	[M + Na]^+^	507, 710, 913	IV(C18:1-OH, Cb)
**1134**	[M + Na]^+^	493, 696, 899	IV(C18:0, Cb, CH_3_)*
**1136**	[M + Na]^+^	- , 712, 915	IV(C18:0-OH, Cb)
**1142**	[M + Na]^+^	515, 718, 921	IV(C20:3, Cb)
**1144**	[M + Na]^+^	517, 720, 923	IV(C20:2, Cb)
**1146**	[M + Na]^+^	519, 722, 925	IV(C20:1, Cb)
**1162**	[M + H]^+^	493, 678, 738, 881, 941, 1102	IV(C20:3, Cb, OAc)
**1162**	[M + Na]^+^	493, 678, 738, 881, 941, 1102	IV(C18:0, Cb, OAc)
**1162**	[M + Na]^+^	535, 738, 941	IV(C20:1-OH, Cb)
**1184**	[M + Na]^+^	515, 700, 760, 903, 963, 1124	IV(C20:3, Cb, OAc)
**1186**	[M + Na]^+^	517, 702, 762, 905, 965, 1126	IV(C20:2, Cb, OAc)
**1188**	[M + Na]^+^	519, 704, 764, 907, 967, 1128	IV(C20:1, Cb, OAc)
**1190**	[M + Na]^+^	521, 766, 969	IV(C20:0, Cb, OAc)
**HAMBI 3275**			
**1092**	[M + Na]^+^	465, 668, 871	IV(C16:0, Cb)
**1096**	[M + H]^+^	469, 672, 875	IV(C18:1, Cb)
**1098**	[M + H]^+^	471, 674, 877	IV(C18:0, Cb)
**1114**	[M + Na]^+^	487, 690, 893	IV(C18:3, Cb)
**1116**	[M + Na]^+^	489, 692, 895	IV(C18:2, Cb)
**1118**	[M + Na]^+^	491, 694, 897	IV(C18:1, Cb)
**1120**	[M + H]^+^	493, 696, 899	IV(C20:3, Cb)
**1120**	[M + Na]^+^	493, 696, 899	IV(C18:0, Cb)
**1122**	[M + H]^+^	495, 698, 901	IV(C20:2, Cb)
**1142**	[M + Na]^+^	515, 718, 921	IV(C20:3, Cb)
**1144**	[M + Na]^+^	517, 720, 923	IV(C20:2, Cb)
**1146**	[M + Na]^+^	519, 722, 925	IV(C20:1, Cb)
**1148**	[M + Na]^+^	521, 724, 927	IV(C20:0, Cb)
**1160**	[M + Na]^+^	533, 736, 939	IV(C20:2-OH, Cb)
**1162**	[M + Na]^+^	535, 738, 941	IV(C20:1-OH, Cb)
**1164**	[M + Na]^+^	537, 740, 943	IV(C20:0-OH, Cb)
**1172**	[M + Na]^+^	545, 748, 951	IV(C22:2, Cb)

*Has methyl group on reducing terminal position.

Similar ESI- and MALDI-MS analyses of the HPLC fractions obtained on purification of the LCOs from the *noeT* mutant strain HAMBI 3275, exhibited [M + H]^+^ peaks at *m/z* 1096, 1098, 1120 and 1122 along with [M + Na]^+^ peaks at *m/z* 1092, 1114, 1116, 1118, 1120, 1142, 1144, 1146, 1148, 1160, 1162, 1164 and 1172 (Table 2). While intense signals were obtained for the LCOs from the mutant strain HAMBI 3275, none of the species corresponds to an acetyl-bearing LCO. One of the most intense ions observed on analysis of the *noeT* mutant strain was at *m/z* 1118 ([M + Na]^+^), which fragments to give B_1_, B_2_ and B_3_ ions at *m/z* 491, 694 and 987 respectively (Additional file 1: Figure S7); the *m/z* increment between the B_1_ and B_2_ ions is 203 (for this and all the mutant strain LCOs), corresponding to a GlcNAc residue without the additional acetyl moiety, and there was no evidence in any of the product ion spectra for the loss of acetic acid, seen so clearly in the spectra of the acetylated LCOs from the wild type strain (Additional file 1: Figure S6). The B_1_ ion at *m/z* 491 corresponds to the presence of a carbamoyl moiety and a C18:1 acyl chain on the non-reducing-terminal residue. From these data, it is evident that the wild type *N. galegae* produces LCOs that bear an acetyl residue on the GlcNAc residue adjacent to the non-reducing residue and that this acetyl group is absent from the LCOs produced by the *noeT* mutant. The nature of the acetyl linkage was demonstrated by treatment of O-acetylated LCO-containing HPLC fractions with mild basic conditions which cleave ester linkages. MALDI-MS analysis of the HPLC fractions following base treatment revealed a reduction of 42 *m/z* units of the protonated and sodiated molecules, and, on product ion analysis, from the relevant fragment ions (Table 3). The data are consistent with the removal, on mild base treatment, of an ester-linked acetyl moiety from the residue adjacent to the non-reducing terminus, whilst the amide-bound fatty acid remained in place as expected. Thus, the acetyl moiety present on the LCOs of *N. galegae* HAMBI 1174 and absent in those from the *noeT* mutant is shown to be ester bound.

**Table 3:** **Effects of de-** ***O*** **-acetylation on the LCOs of HAMBI 1174**

Precursor ion			Fragment Ions		Structure Assignment	
Before base treatment	After base treatment	Molecular Species	Before base treatment	After base treatment	Before base treatment	After base treatment
1162	1120	[M + H]^+^	493, 678, 738, 881, 941, 1102	493, 696, 899	IV(C20:3, Cb, OAc)	IV(C20:3,Cb)
1184	1142	[M + Na]^+^	515, 700, 760, 903, 963, 1124	515, 718, 921	IV(C20:3, Cb, OAc)	IV(C20:3, Cb)
1186	1144	[M + Na]^+^	517, 702, 762, 905, 965, 1126	517, 720, 923	IV(C20:2, Cb, OAc)	IV(C20:2, Cb)
1188	1146	[M + Na]^+^	519, 704, 764, 907, 967, 1128	519, 722, 925	IV(C20:1, Cb, OAc)	IV(C20:1, Cb)

## Discussion

In this study, the genome sequences of two *N. galegae* strains is reported; the type strain HAMBI 540^T^ (symbiovar orientalis) and strain HAMBI 1141 (symbiovar officinalis). The genome sequences revealed a previously unrecognized *nod* gene, *noeT*, in close vicinity of the known symbiosis genes.

Nod factors of rhizobia other than *N. galegae* have been found to have acetyl moieties substituted on the terminal GlcNAc residues, the function encoded by genes *nodL*, *nodX* and *nolL*. The *nodL* gene has been shown to introduce one *O*-acetyl moiety at the C-6 position of the non-reducing-terminal GlcNAc residue in *R. leguminosarum* [28, 29]. The ability of *R. leguminosarum* sv. viciae strain TOM to nodulate cv. Afghanistan pea is dependent on the product of the *nodX* gene, which is required for *O*-acetylation of the C-6 of the reducing-terminal residue of the GlcNAc backbone of Nod*Rlv*-V(Ac, C_18:4_) [30]. However, evidence has also been found that *nodX* can be functionally replaced by *nodZ*, producing a NF that is fucosylated on the reducing-terminal residue [31]. A third type of acetyl transferase involved in modifying Nod factors is NolL, which *O*-acetylates the C4 position of the fucose residue located on the reducing-terminal backbone residue in NFs of *Mesorhizobium loti* (formerly *Rhizobium loti*) [32], *R. etli* [33] and *S. fredii* NGR234 (formerly *Rhizobium* sp. NGR234) [34].

The *noeT* gene of *N. galegae* is highly similar to the *hsnT* gene in *R. leguminosarum* sv. trifolii ICC105 (accession number EU919402). There is no published evidence for the function of this *hsnT* gene, which is assigned a putative acetyl transferase function. Since the *noeT* gene is located in the symbiosis gene region and has a nod-box, the homology with *hsnT* indicated that this could be the acetyltransferase gene involved in *N. galegae* Nod factor biosynthesis. Recently, *hsnT* genes homologous to the *R. leguminosarum* sv. trifolii ICC105 *hsnT* gene have been revealed in other rhizobial strains: *R. tropici* CIAT 899 [22] (protein YP_007336040), *R. tropici* WUR1 [35] (protein AFJ42562) and *R. grahamii* CCGE 502 [36] (protein WP_016558512). No function has, however, yet been described for the *hsnT* genes in these strains. The NFs of CIAT 899 have been analysed [37]– [39], but no acetyl substituent has been reported in the same position as in *N. galegae*. The acetyl substituent of *N. galegae* is in a very unusual position, on the GlcNAc residue adjacent to the non-reducing-terminal residue, while Nod factors of CIAT 899 are acetylated on the non-reducing-terminal residue. Given the unusual position of the acetyl substituent, it was for a long time thought to be important for the very strict host specificity of *N. galegae*. To date, the only strains known to produce NFs modified in the same position are *M. loti* NZP2213 which has a fucose in this position [9, 40], and *Mesorhizobium* sp. strain N_33_ (*Oxytropis arctobia*) and *Rhizobium* sp. BR816 (broad-host range strain isolated from *Leucaena leucocephala*) which can bear an acetyl substituent in the same position [9, 41, 42]. The fucose residue of NZP2213 does not appear to extend or limit host-range specificity in comparison to other *M. loti* strains which lack NFs with this modification, and it was thus suggested to provide protection of the NF against degradation or to be an adaptation to a particular as yet unidentified host-specific receptor [40]. No specific biological function for the acetyl substituent on the nonterminal GlcNAc residue has been reported for strain BR816, nor has any functional gene been assigned in this strain. However, there is high sequence similarity between NoeT of *N. galegae* and a pair of hypothetical proteins in BR816 (WP_018240294 and WP_01824095). When compared to *nodL*, *nodX* and *nolL* genes, the *N. galegae* acetyltransferase gene shows highest similarity to TOM *nodX*, with 42% positives (27% identity) over a 318 residues long alignment (out of 639 residues in *N. galegae*).

Mass spectometric analysis of the NFs of the wild type strain HAMBI 1174 and the *noeT* deletion mutant HAMBI 3275 revealed LCO structures that differ from those reported by Yang and associates (1999) [11] in backbone length and fatty acyl substitution. In the previous work, strains overexpressing the *nod* genes were used, which might have caused the structural difference in the fatty acyl chain [43]. In addition, we have here detected a methylated LCO among HAMBI 1174 NFs (Table 2). The methyl group is located on the reducing-terminal position, another rare position for NF substitutions [9]. The genetic determinants for this substitution are, however, unknown. Nevertheless, in this study, mass spectrometric analysis clearly showed that the NFs of the mutant strain lack the acetyl substituent on the GlcNAc residue adjacent to the non-reducing-terminal residue, while a majority of the wild-type NFs are acetylated. This is consistent with the assumption that *noeT* encodes a protein that is responsible for the addition of this acetyl moiety to the LCO. Nod factors of the sv. officinalis strain HAMBI 1207, a derivative strain of HAMBI 1141, have been shown to be identical to those of symbiovar orientalis [11], indicating that this gene probably has the same function in both symbiovars. Plant experiments showed that deletion of this gene does not affect the ability of *N. galegae* HAMBI 1174 to induce effective nodules on *G. orientalis*, showing that *noeT* alone is not directly responsible for the host specific nodulation of *N. galegae*. Furthermore, *R. tropici* CIAT 899, containing a homologous protein [22], was not able to form nodules on *G. orientalis* when tested in our laboratory. The inability of the mutant strain to induce nodules on *T. repens*, *P. sativum* cv. Afghanistan, *P. vulgaris*, *V. hirsuta* or *A. sinicus* also indicates that the presence of the acetyl moiety is not the reason, or at least not the sole reason, behind the inability of *N. galegae* to nodulate these plant species.

Even though initiation of symbiosis was not affected by the altered NF structure in the mutant, and nodules were formed at an equal rate for both wild type and mutant strains during the first two weeks post inoculation (Figure 7), the final number of nodules was significantly lower for the mutant compared with the wild type at the end of the experiment. It has been suggested that Nod factor substitutions can protect NFs against degradation by plant chitinases [44, 45]. One possible explanation for the mutant phenotype observed in the plant experiment of this study is that the acetyl moiety on the *N. galegae* NF might provide a protective function against degradation. This concept was also suggested previously [41]. Assuming that the concentration of NF-degrading compounds increases with time, this could explain why nodule formation on plants inoculated with the mutant strain stagnates after 16 days post-inoculation (Figure 7). The same role has been suggested for the fucose residue present in the corresponding position on the *M. loti* NZP2213 LCO [40]. Nevertheless, the possibility that *noeT* is important for host specificity under conditions not tested here must not be excluded.

### Are all genes in the symbiosis gene region coupled to functions in symbiosis?

In addition to the *noeT* gene, there are some other genes in the symbiosis gene region that deserve attention. The symbiosis gene regions of the sequenced strains share the same complement of genes with one exception, an additional sigma factor gene, *rpoN2*, found in HAMBI 540^T^. Many genes in block 2 of Figure 5 are regulated by NifA and RpoN [46]. In *R. etli*, there is evidence that separate *rpoN* genes are involved in regulation under free-living and symbiotic conditions [47]. The two *rpoN* genes in HAMBI 540^T^ have 87% amino acid identity, but only 41% over the first 41 amino acids. In *Rhodobacter sphaeroides*, this region of *rpoN* (region I) has been shown to be important for promoter recognition and for interaction with the activator protein [48]. The *rpoN2* gene of HAMBI 540^T^ is preceded by a predicted hypothetical gene (RG540_PA10540, Figure 5) containing a TRX family domain. However, this gene does not have any significant similarity to the NifA-regulated peroxiredoxin genes found upstream of *rpoN* in the symbiosis regions of *R. etli* [46, 47]. Thus, studies need to be conducted to investigate if *rpoN2* in HAMBI 540^T^ is involved in regulation of symbiosis genes, and to determine if this gene contributes to the difference in nitrogen fixation observed between strains of symbiovars orientalis and officinalis.

*N. galegae* also has two versions of the C_4_-dicarboxylate carrier protein-coding gene *dctA*: one on the chromosome and one in the symbiosis gene region downstream of *nifQ* (Figure 5). Results of GenBank searches indicate that the genomic context of *nifQ* followed by *dctA* is common among strains of *S. fredii*. There are, however, conflicting data as to whether the second copy of *dctA* is essential or not for symbiotic nitrogen fixation [49, 50]. A possible explanation for the extra copy of *dctA* in the symbiosis gene region might be that it leads to a more efficient energy intake at times when the symbiosis genes are expressed. The NifQ protein in *N. galegae* has, on the other hand, diverged remarkably from NifQ proteins in other rhizobia, even lacking the molybdenum-binding motif. Analysis of the ratio of nonsynonymous/synonymous substitution rates showed that *nifQ* has a relatively higher rate of nonsynonymous substitutions than any other gene in the symbiosis gene region (Figure 6). Analyses of the evolution of *N. galegae nifQ* in relation to other rhizobial species indicated that the evolution of this gene is not due to positive selection but a higher level of nonsynonymous mutations. This and the fact that the molybdenum-binding motif is missing from *nifQ* indicates that this gene is possibly nonessential for *N. galegae*, and is most probably a nonfunctional pseudogene. At this point, there is no evidence that *nifQ* is functional in *N. galegae*.

The *nodO* gene is located immediately downstream of *dctA*. NodO is a calcium-binding protein which is exported to the growth medium without cleavage of the N-terminal region [51]. Based on the location of the T1SS genes, directly downstream of *nodO* (Figure 5), together with their similarity to the *prsDE* genes previously found to be responsible for NodO secretion in *R. leguminosarum* [52], it seems probable that these two genes are responsible for transporting the NodO protein out of the cell. This might, however, be a *N. galegae*-specific system, because BLAST alignments showed that the fragment used by Tas *et al*. to design species-specific PCR primers for *N. galegae* [53] originates from the T1SS gene RG540_PA10320. The arrangement of T1SS genes directly downstream of *nodO* is different from the arrangement in many other *nodO*-containing rhizobia, where the main genes responsible for NodO secretion mainly seem to be located distantly from the *nodO* gene itself [51, 52]. Many T1SSs require a third protein in the form of an outer membrane protein to function, but in *N. galegae* no such ORF is found downstream of the two T1SS genes. Similarly, there was no OMP identified in the *prsDE* system of *R. leguminosarum* [52], although the authors speculated that a protein that is not linked to the *prsDE* genes is contributing to NodO secretion. The *nodO* gene has been shown to compensate for mutations in *nodFE* of *R. leguminosarum* sv. viciae in nodulation of vetch and of pea, although restoration of nodulation of pea requires *nodL* in addition to *nodO* when *nodFE* is not present [54]. In addition, *nodO* from *Rhizobium* sp. BR816 suppressed the nodulation defect of *S. fredii* NGR234 and *R. tropici* CIAT 899 *nodU* mutants on the host plant *L. leucocephala* [55]. NodO has also been reported to have an effect the host range of certain rhizobia [55, 56]. Sutton *et al*. [57] proposed that the cation fluxes across the plasma membrane induced by NodO may amplify the response induced by NFs. Perhaps *nodO* can also compensate for the *noeT* mutation in *N. galegae*?

### Secretion systems may play a role in symbiosis

The presence of a third replicon in HAMBI 1141 was determined previously [1], but now we can confirm that this additional plasmid is an important part of the genome. The fact that genes required for symbiosis are held on the plasmid, together with genes for conjugative transfer is interesting from an evolutionary perspective. Experiments performed in this work showed that *N. galegae* sv. officinalis strain HAMBI 1207 was able to transfer its symbiosis plasmid to the *nod* mutant sv. orientalis strain HAMBI 1587. However, transconjugants were observed only among cells from a selective plate where exconjugants were plated without dilution. This indicates that the transfer frequency might be very low, a conclusion that is also supported by the fact that no true transconjugants were found when HAMBI 1141 was mated with *A. fabrum* strain C58C1 and the *nod* mutant strains *S. meliloti* HAMBI 1213 and *R. leguminosarum* sv. viciae HAMBI 1594. These strains have previously been shown to induce root nodules on the hosts *Medicago sativa* (*A. fabrum* and *S. meliloti*) and *Vicia villosa* (*R. leguminosarum*) when complemented with a cosmid clone containing common *nod* genes of *N. galegae* HAMBI 1174 [58]. It is also not possible to exclude a scenario where donor cells were still present on the selective plate, so that conjugation may have taken place only at the inoculation stage, in the presence of the plant. Regulation of *traR* transcription activator gene expression, and thereby regulation of *tra* gene expression, by plant metabolites has been suggested for *S. fredii* strain NGR234, which has T4SS genes homologous to those found on pHAMBI1141b on its plasmid pSfrNGR234a [59]. Nevertheless, when conjugation was attempted between HAMBI3490 and HAMBI 1587 it resulted in the absence of transconjugants irrespective of whether the cells were diluted or not prior to selection for the recipient. This indicates that the lack of transfer was not due to low transfer frequency, but rather the lack of the T4SS genes on the chromid. The inability of HAMBI 1141 to transfer its symbiosis plasmid to the corresponding plasmid-cured strain indicates that there is an additional factor restricting plasmid transfer between *N. galegae* strains that differ only in the presence of the symbiosis plasmid. However, the results obtained in this study indicate that despite the presence of all necessary genes for a self-transmissible plasmid, the symbiosis plasmid in HAMBI 1141 is not self-transmissible. It remains to be shown whether some of these genes are in fact nonfunctional. However, it can be noted that type IV secretion in *N. galegae* is most likely not involved in directing symbiosis, because no *nod* boxes have been found upstream from the T4SS operons.

Type VI secretion, on the other hand, has been reported to be important for symbiosis-related functions in nitrogen fixation: *R. leguminosarum* strain RBL5523, which normally induces ineffective nodules on pea, gained the ability to induce effective nodules when the *imp* operon was mutated [60]. The T6SS found in HAMBI 540^T^ might also contribute to its host specificity, considering that this feature is not found in the sv. officinalis strain HAMBI 1141. Future work will shed light on the role of the T6SS in strain HAMBI 540^T^.

## Conclusions

This study demonstrates that despite the distinct symbiotic properties, there is a high degree of genomic similarity between the two symbiovars of *N. galegae*, represented by strains HAMBI 540^T^ and HAMBI 1141. The availability of the genome sequences will be invaluable for future research on *N. galegae*. The results of this work also showed that, based on the number of shared orthologous genes and genomic alignments, *N. galegae* is more closely related to *R. leguminoarum* sv. viciae 3841 than to *A. fabrum* C58, *S. meliloti* 1021, *R. tropici* CIAT 899 or *S. medicae* WSM419. In addition, we report for the first time the gene responsible for acetylation of the GlcNAc residue adjacent to the non-reducing-terminal residue on the *N. galegae* Nod factors. We have named this gene *noeT*, a name reflecting the function involved in shaping the Nod factor albeit not directly determining host specificity as the gene name *hsnT* implies. We have demonstrated that the *noeT* gene alone is not essential for nodulation of *Galega* plants, but we speculate that it might have a protective effect on the Nod factor of *N. galegae*. We have also shown that the symbiosis plasmid of HAMBI 1141 is conjugative, although it does not seem to be self-transmissible.

## Methods

### Bacterial strains and growth conditions

Strains and plasmids used in this study are described in (Additional file 2: Table S1). *N. galegae* strains HAMBI 540^T^, HAMBI 1141 and HAMBI 1174 and *R. tropici* strain CIAT 899 (HAMBI 1163) were obtained from the HAMBI culture collection (University of Helsinki, Faculty of Agriculture and forestry, Division of Microbiology and Biotechnology). The rhizobial strains were grown on TY or YEM agar plates and in TY broth. Culture media of HAMBI 1174 and its *noeT* mutant were supplied with spectinomycin (500 μg/mL) and neomycin (25 μg/mL) respectively. *E. coli* strains used for mutant construction were grown in LB media. Media were supplied with appropriate antibiotics: streptomycin 30 μg/mL, spectinomycin 50 μg/mL, gentamicin 25 μg/mL, kanamycin 50 μg/mL.

### DNA isolation

Total DNA of strains HAMBI 540^T^ and HAMBI 1141 was isolated using a CTAB (hexadecyltrimethylammonium bromide) procedure modified from Wilson (1994) [61] (see Additional file 2 for detailed description). Plasmid DNA was isolated using the GeneJET Plasmid Miniprep Kit (Thermo Scientific). DNA for PCR verification of clones was isolated using the PrepMan Ultra Sample Preparation Reagent (Applied Biosystems), applying the protocol for preparation of samples for bacterial and fungal testing from culture broths.

### Genome sequencing, assembly and annotation

A library was constructed from HAMBI 540^T^ and HAMBI 1141 DNA and sequenced on a Genome Sequencer FLX Titanium (Roche). The obtained sequences were assembled using Newbler (Roche). One mate-pair library (1.5 – 3.5 kb) for each strain was constructed using the SOLiD mate-pair library kit and sequenced on a SOLiD4 Sequencer (Life Technologies). The obtained sequences were used for scaffolding and correction of homopolymer errors in the 454 contigs. PCR and Sanger sequencing was used for closing of the gaps in the scaffolds. The final closure was done using long reads obtained from two SMRT cells for both genomes run on a PacBio RS (Pacific Biosciences) (see Additional file 2 for detailed description).

Gene prediction was done with Prodigal ver. 2.50 [62] as part of the PANNZER annotation pipeline (Koskinen *et al*. unpublished). The tRNA genes were annotated using tRNAscan-SE 1.3.1 [63] and rRNA genes identified with RNAmmer 1.2 [64]. The gene predictions were manually checked using the Artemis software [65]. To ascertain the validity of the third chromid criterion (core genes found on the chromosome in other species) in *N. galegae*, the blastp service was used to determine homology of the protein sequences of the genes on the chromids (as predicted by Prodigal) to the set of 280 core genes of 69 taxa initially used to define chromids [12], with a threshold of minimum 70% identity. A complete list of the genes in the genomes is provided in an additional file (see Additional file 3). The genome sequences were submitted to the European Nucleotide Archive: [EMBL:HG938355-HG938357] and [EMBL:HG938353-HG938354]. The sequences can be accessed through the links http://www.ebi.ac.uk/ena/data/view/HG938353-HG938354 (HAMBI 540^T^) and http://www.ebi.ac.uk/ena/data/view/HG938355-HG938357 (HAMBI 1141).

### Bioinformatics analyses

The two sequenced genomes were aligned using the genome alignment software progressiveMauve [66], using default options. Genes were assigned to COG categories by an RPS-BLAST search (as part of the NCBI toolbox) against the COG collection [67] in the conserved domain database [68]. The OrthoMCL software [69] was used to find ortholog groups between the two strains of *N. galegae* and related rhizobial strains. The software was run with default settings. Custom Perl, Python and Biopython [70] scripts were used to modify output from these analyses and to create data files for Circos [71], which was used to make circular data representations of the genomes. A structural genomics analysis comparing the two *N. galegae* genomes with the complete genomes of strains *R. leguminosarum* sv. viciae 3841, *R. tropici* CIAT 899, *S. medicae* WSM419 and *S. meliloti* 1021 was generated using the megablast algorithm [72], retaining only hits with an alignment length of at least 1000 bp. These results were visualised with Artemis Comparison Tool (ACT) [73]. The reference genomes used for alignments of pHAMBI1141b and related rhizobial strains are listed in Additional file 2: Table S2). CodonW [74] was used for analysis of total codon usage. For this analysis chromosomal genes only were used. Hypothetical genes with no sequence identity with other proteins were removed from the data set, as well as transposon- and phage-related genes and genes shorter than 50 aa. A description of the analysis of evolutionary history of the RepABC systems and the analyses of substitution rates and positive selection in the *nifQ* gene is provided in Additional file 2.

### noeT mutant construction and Nod factor analysis

A *noeT* gene replacement mutant (strain HAMBI 3275) was constructed in HAMBI 1174 to study the function of this gene in symbiosis. Nod factors of this mutant and its wild-type parental strain were extracted and analysed by mass spectrometry. The effect of the mutation on nodule formation and nitrogen fixation was assessed through plant inoculation assays on the original host plant, *G. orientalis*. The mutant strain was also tested on host plants of other rhizobial strains to check whether the mutation had an effect on host range. The methods for mutant construction, Nod factor extraction, mass spectrometric analysis and plant assays are described in detail in Additional file 2.

### HAMBI 1141 symbiosis plasmid conjugation tests

In order to study whether the symbiosis plasmid of strain HAMBI 1141 is conjugative, conjugation tests were performed between HAMBI 1141 or its streptomycin-resistant derivative strain HAMBI 1207 and nodulation defective strains of both symbiovars of *N. galegae*, *S. meliloti*, *R. leguminosarum* and *A. fabrum*. A plasmid-cured derivative of HAMBI 1207 as well as a HAMBI 1141 deletion mutant lacking the T4SS gene region on the chromid, were constructed to study the impact of the chromid-borne T4SS genes on conjugation of the symbiosis plasmid. Biparental matings, plant tests and transconjugant confirmation was performed as described in Additional file 2.

### Availability of supporting data

The data sets supporting the results of this article are included within the article and its additional files. The complete genome sequences of *N. galegae* strains HAMBI 540^T^ and HAMBI 1141 are publicly available in the European Nucleotide Archive with accession numbers HG938353-HG938354 (HAMBI 540^T^) and HG938355-HG938357 (HAMBI 1141) (http://www.ebi.ac.uk/ena/data/view/HG938353-HG938354, http://www.ebi.ac.uk/ena/data/view/HG938355-HG938357). Accession numbers of reference sequences used are included in Additional file 2.

## Electronic supplementary material

Additional file 1: Figure S1: ML phylogeny of concatenated RepABC protein sequences from *N. galegae* strains HAMBI 540^T^ and HAMBI 1141, and 12 strains representing *Rhizobium*, *Sinorhizobium*, *Mesorhizobium* and *Agrobacterium*. Figure S2: A figure illustrating the location of singletons of *N. galegae* strains HAMBI 540^T^ and HAMBI 1141 identified in the OrthoMCL analysis with 5 genomes. The inner ring shows the COG categories of these singletons. Figure S3: A figure illustrating the location of orthologous genes of *N. galegae* strains HAMBI 540^T^ and HAMBI 1141. Orthologs between any gene in HAMBI 1141 and a gene located on the chromosome of HAMBI 540^T^ are shown with a blue link, while orthologs located on any replicon in HAMBI 1141 and on the chromid of HAMBI 540^T^ are shown in green. Figure S4: A Maximum likelihood tree of rhizobial *nifQ* sequences. This tree was used for PAML analyses, without branch lengths. Figure S5: RP-HPLC fractionation of *N. galegae* HAMBI 1174 and *noeT* mutant strain LCO-containing SPE fractions a) HAMBI 1174 (45% SPE fraction) b) HAMBI 1174 (60% SPE fraction), c) *noeT* mutant (45% SPE fraction) d) *noeT* mutant (60% SPE fraction). Regions 2 and 3 correspond to known elution positions for LCOs. The region marked 1 was shown to contain a strongly UV-absorbing polymeric contaminant – no LCOs were detected in this region. Figure S6: CID product ion spectrum of the LCO from wild type strain *N. galegae* HAMBI 1174 giving an [M + H]^+^ ion at *m/z* 1162, eluting with retention time 46 minutes. Figure S7: CID product ion spectrum of *N. galegae* HAMBI 1174 *noeT* mutant strain NF at *m/z* 1118 ([M + Na]^+^) at retention time 45 minutes. (PDF 356 KB)

Additional file 2: **This text file contains a detailed description of the materials and methods used in this study.** Table S1 Strains and plasmids used in this study. Table S2 Accession numbers of reference genomes used. Table S3 Accession numbers of RepABC sequences used. Table S4 Primers used in this study. (PDF 793 KB)

Additional file 3: **Lists of all genes predicted in the genomes of** ***N. galegae*** **strains HAMBI 540** ^**T**^ **and HAMBI 1141.** (XLSX 580 KB)

## Abbreviations

NF: Nod factor
LCO: Lipochitin oligosaccharide
GlcNAc: β-1,4-linked N-acetylglucosamine
COG: Clusters of Orthologous Groups
EPS: Exopolysaccharide
LPS: Lipopolysaccharide
Flp/Tad: Fimbrial low-molecular-weight protein/tight adherence protein
T1SS: Type I secretion system
IS: Insertion sequence
ABC: ATP binding cassette
LRT: Likelihood ratio test
ML: Maximum likelihood
RP-HPLC: Reversed phase high-performance liquid chromatography
SPE: Solid phase extraction
ESI-MS: Electrospray ionization mass spectrometry
MALDI-MS: Matrix-assisted laser desorption/ionization mass spectrometry
CID: Collision-induced dissociation
OMP: Outer membrane protein.
