# Supplementary material for: Genome sequencing of two Neorhizobium galegae strains reveals a noeT gene responsible for the unusual acetylation of the nodulation factors
Source: BMC Genomics. 2014 Jun 19;15(1):500. doi: 10.1186/1471-2164-15-500 (PMC4085339; doi:10.1186/1471-2164-15-500)
Supplement: Supplementary file 1 — Additional file 1: Figure S1: ML phylogeny of concatenated RepABC protein sequences from N. galegae strains HAMBI 540T and HAMBI 1141, and 12 strains representing Rhizobium, Sinorhizobium, Mesorhizobium and Agrobacterium. Figure S2: A figure illustrating the location of singletons of N. galegae strains HAMBI 540T and HAMBI 1141 identified in the OrthoMCL analysis with 5 genomes. The inner ring shows the COG categories of these singletons. Figure S3: A figure illustrating the location of orthologous genes of N. galegae strains HAMBI 540T and HAMBI 1141. Orthologs between any gene in HAMBI 1141 and a gene located on the chromosome of HAMBI 540T are shown with a blue link, while orthologs located on any replicon in HAMBI 1141 and on the chromid of HAMBI 540T are shown in green. Figure S4: A Maximum likelihood tree of rhizobial nifQ sequences. This tree was used for PAML analyses, without branch lengths. Figure S5: RP-HPLC fractionation of N. galegae HAMBI 1174 and noeT mutant strain LCO-containing SPE fractions a) HAMBI 1174 (45% SPE fraction) b) HAMBI 1174 (60% SPE fraction), c) noeT mutant (45% SPE fraction) d) noeT mutant (60% SPE fraction). Regions 2 and 3 correspond to known elution positions for LCOs. The region marked 1 was shown to contain a strongly UV-absorbing polymeric contaminant – no LCOs were detected in this region. Figure S6: CID product ion spectrum of the LCO from wild type strain N. galegae HAMBI 1174 giving an [M + H]+ ion at m/z 1162, eluting with retention time 46 minutes. Figure S7: CID product ion spectrum of N. galegae HAMBI 1174 noeT mutant strain NF at m/z 1118 ([M + Na]+) at retention time 45 minutes. (PDF 356 KB) [file 12864_2014_6200_MOESM1_ESM.pdf]

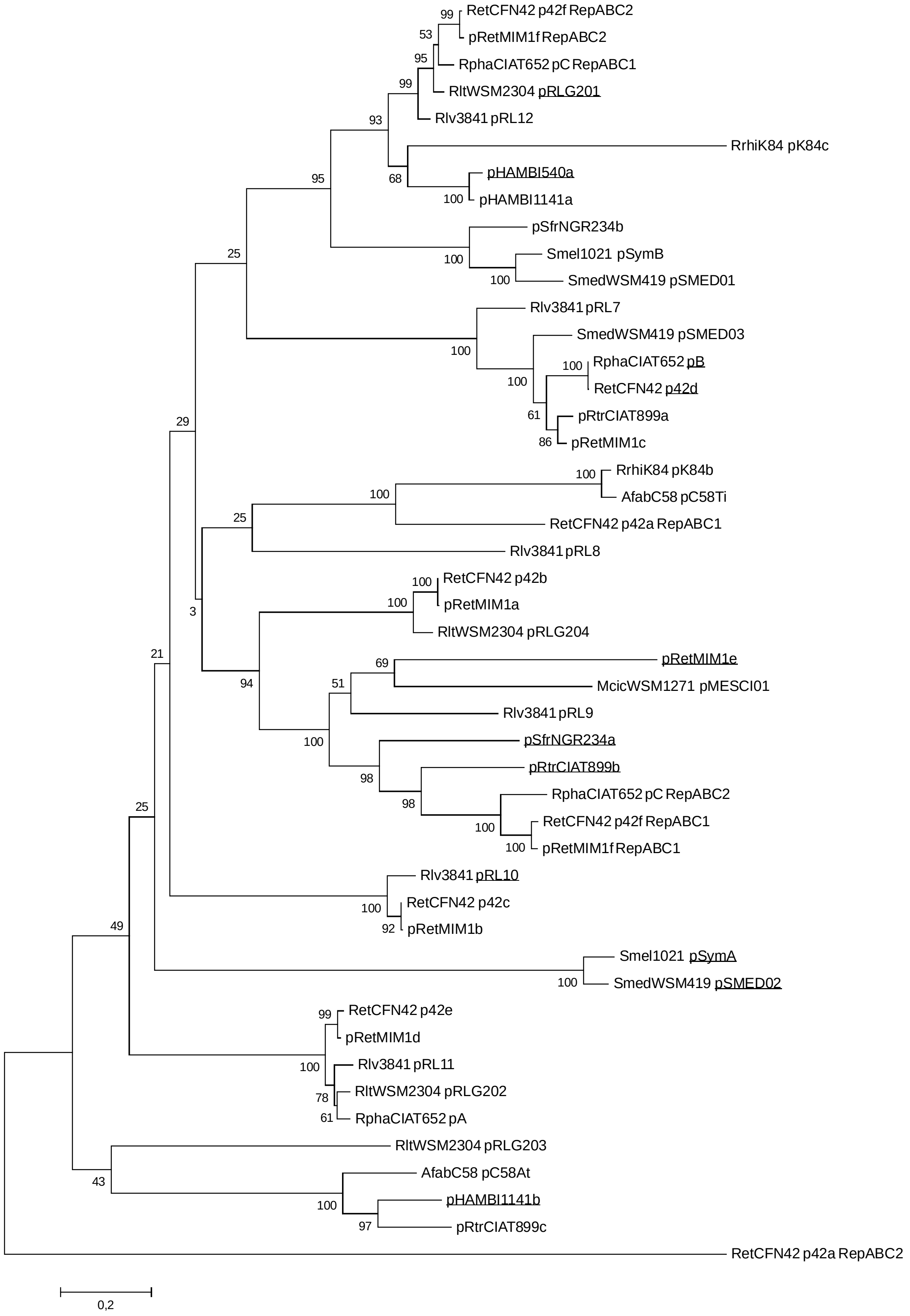

Figure S1. Phylogeny of concatenated RepABC proteins of replicons with this replication system in *N. galegae* strains HAMBI 540[T] and HAMBI 1141, together with 12 strains representing *Rhizobium*, *Sinorhizobium*, *Mesorhizobium* and *Agrobacterium*. For those replicons which have multiple *repABC* operons, the operon is indicated. Replicons containing symbiosis genes are underlined. Rlv3841; *R. leguminosarum* sv. viciae 3841, RltWSM2304; *R. leguminosarum* sv. trifolii WSM2304, RtrCIAT899;*R. tropici* CIAT 899, RphaCIAT652; *R. phaseoli* CIAT 652, RetMIM1; *R. etli* sv. mimosae str. Mim1, RetCFN42; *R. etli* CFN 42, SfrNGR; *S. fredii* NGR234, Smel1021; *S. meliloti* 1021, SmedWSM419; *S. medicae* WSM419, McicWSM1271; *M. ciceri* sv. biserrulae WSM1271, RrhiK84; *R. rhizogenes* K84, AfabC58; *A. fabrum* C58. The evolutionary history was inferred by using the Maximum Likelihood method. Bootstrap values are shown next to the branches.

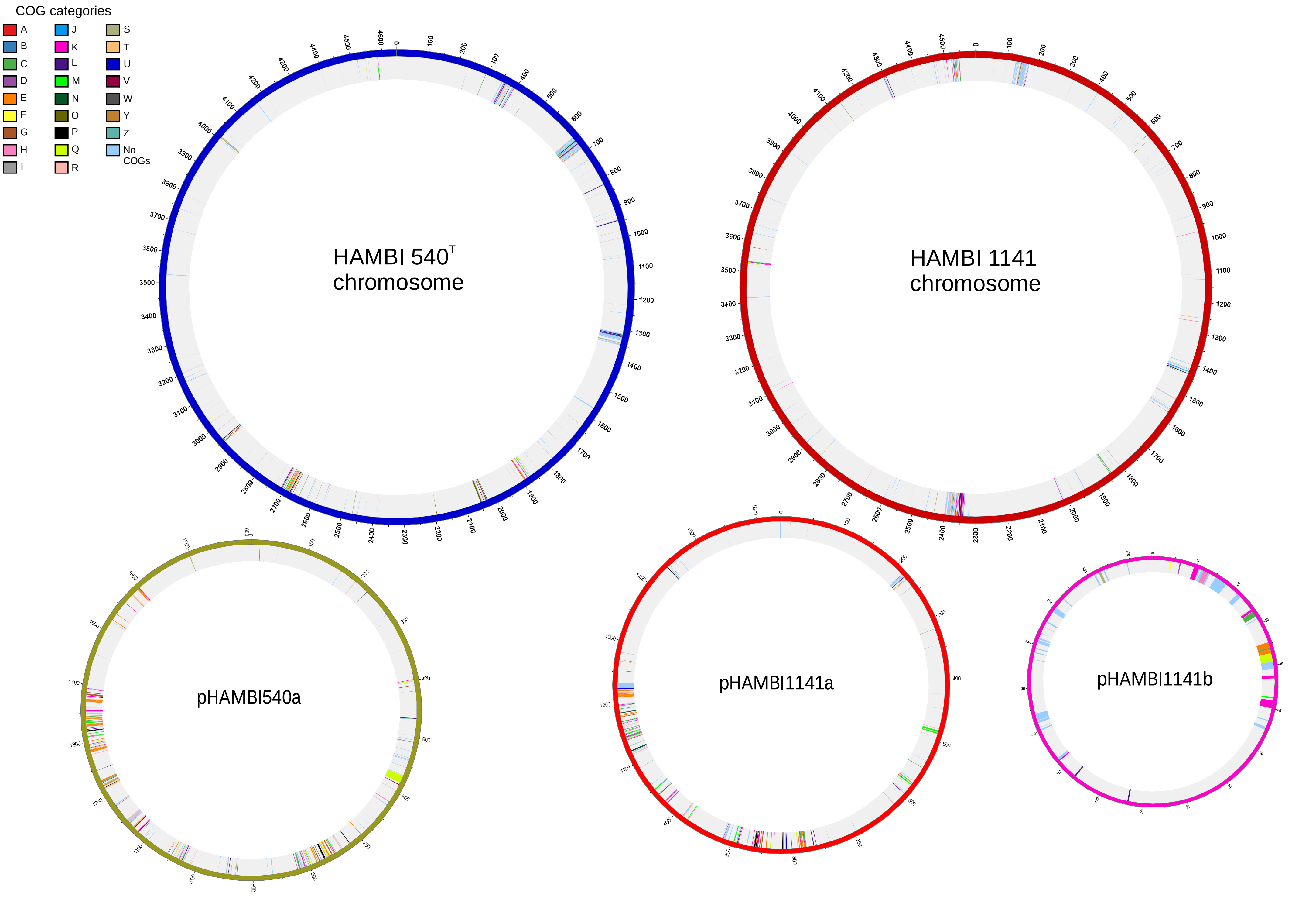

Figure S2. Illustration of the location singletons identified in the genomes of *N. galegae* HAMBI 540[T] and HAMBI 1141 in the OrthoMCL analysis.

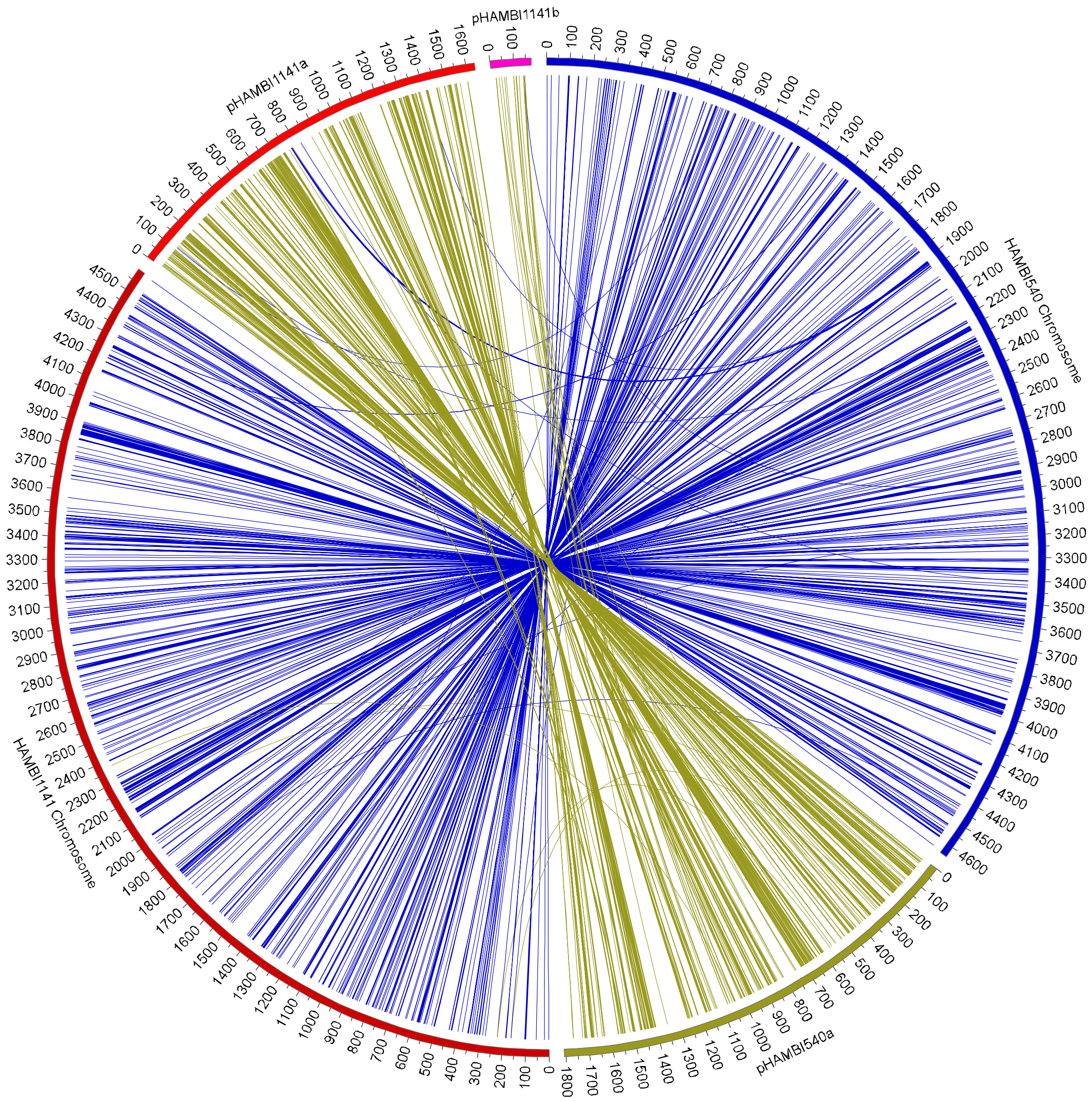

Figure S3. Orthologous genes of *N. galegae* strains HAMBI 540[T] and HAMBI 1141.

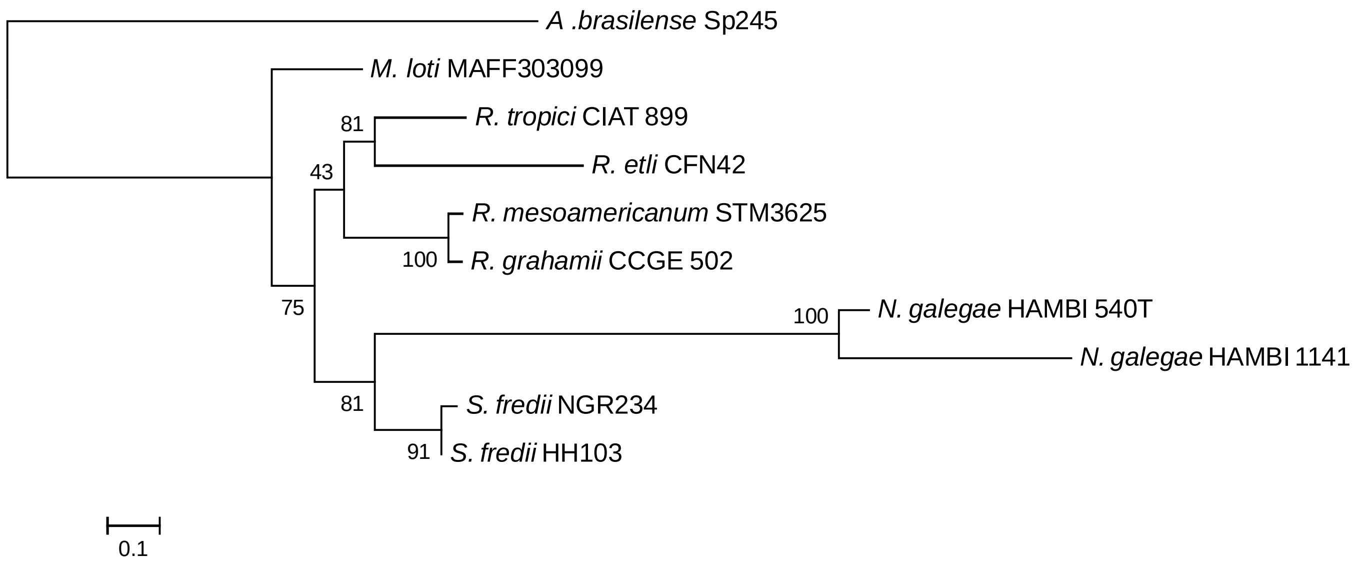

Figure S4. NifQ ML tree used for PAML analyses.

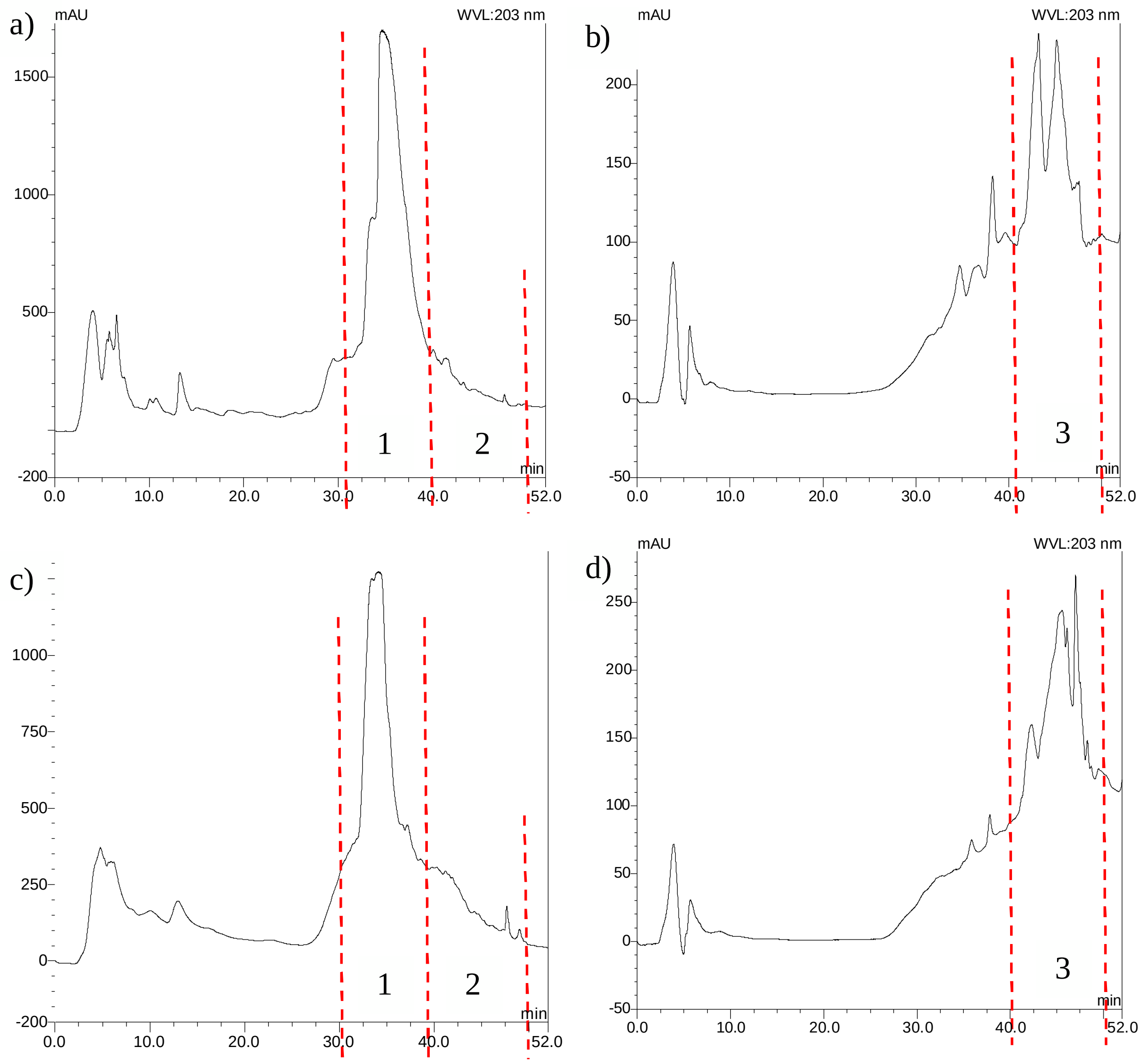

Figure S5. RP-HPLC fractionation of *N. galegae* HAMBI 1174 and *noeT* mutant strain LCO-containing SPE fractions. a) HAMBI 1174 (45 % SPE fraction) b) HAMBI 1174 (60 % SPE fraction), c) *noeT* mutant (45 % SPE fraction) d) *noeT* mutant (60 % SPE fraction). Regions 2 and 3 correspond to known elution positions for LCOs.

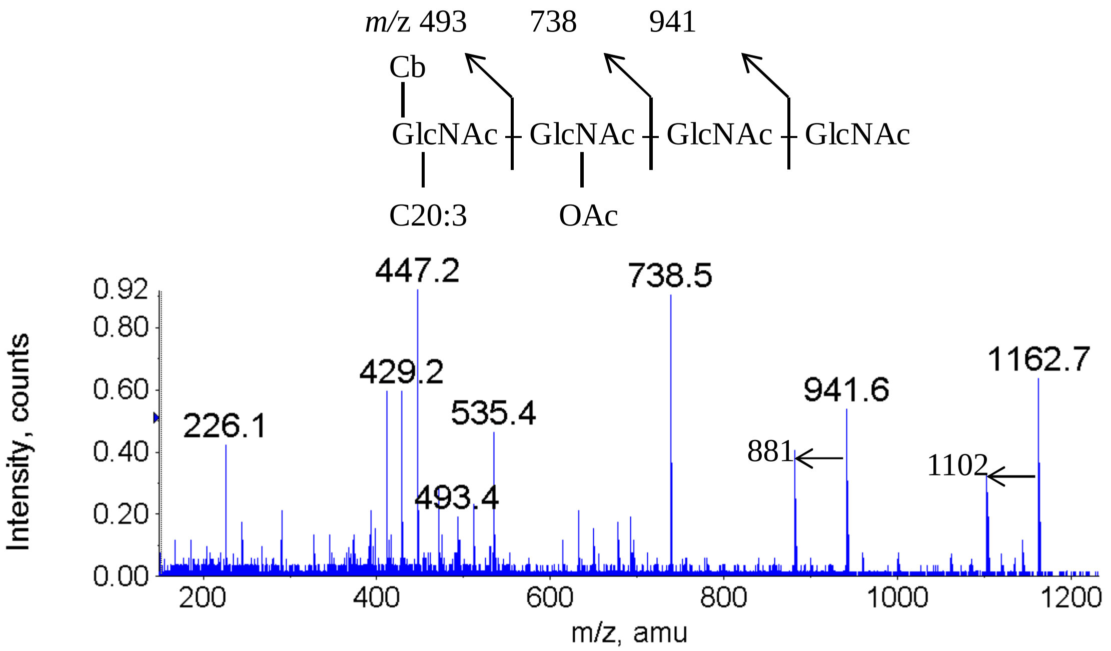

Figure S6. CID product ion spectrum of the LCO from wild type strain *N. galegae* HAMBI 1174

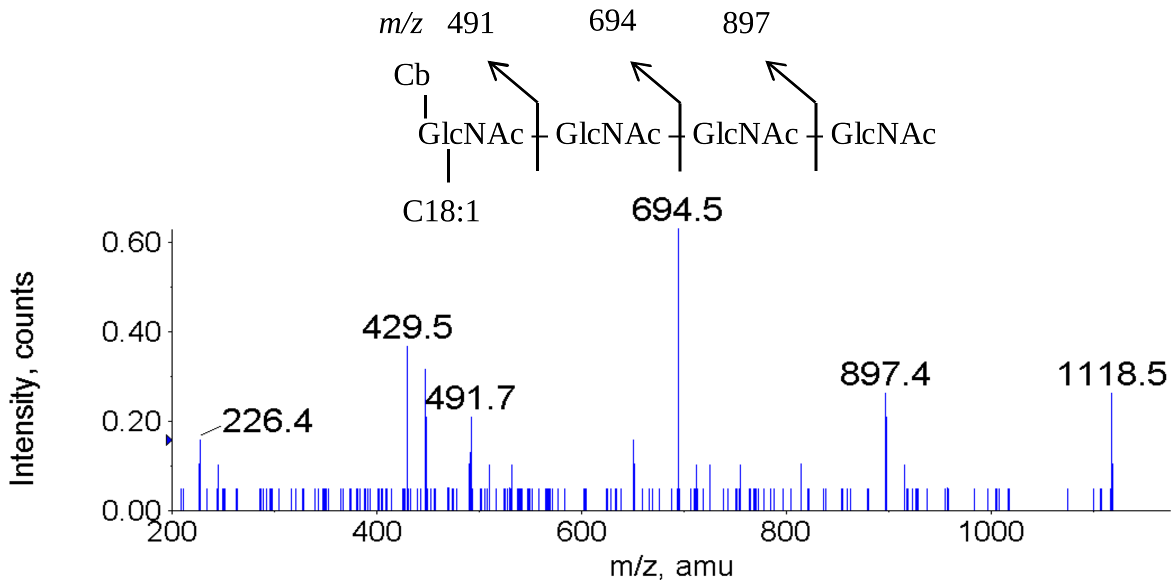

Figure S7. CID product ion spectrum of *N. galegae* HAMBI 1174 *noeT* mutant strain NF
