# Supplementary material for: Genome sequencing of two Neorhizobium galegae strains reveals a noeT gene responsible for the unusual acetylation of the nodulation factors
Source: BMC Genomics. 2014 Jun 19;15(1):500. doi: 10.1186/1471-2164-15-500 (PMC4085339; doi:10.1186/1471-2164-15-500)
Supplement: Supplementary file 2 — Additional file 2: This text file contains a detailed description of the materials and methods used in this study. Table S1 Strains and plasmids used in this study. Table S2 Accession numbers of reference genomes used. Table S3 Accession numbers of RepABC sequences used. Table S4 Primers used in this study. (PDF 793 KB) [file 12864_2014_6200_MOESM2_ESM.pdf]

Additional file 2: Detailed materials and methods

DNA isolation for genome sequencing

Cells were grown to stationary phase and harvested by centrifugation in 40 mL aliquots. Harvested cells were washed and resuspended in 2 mL of 100 mM Tris 50 mM EDTA pH 8.0. Then, 20 µL of 1 M β-mercaptoethanol was added, followed by 200 µl of 10 % SDS. Tubes were inverted to mix and cells were lysed for 5 min. Next, 200 µl of 5 M NaCl was added and tubes incubated at 65 °C for 10 min, thereafter 875 µL of 5 M KAc (3 M K^+/5 M Ac^- pH 5.5) was added and the tubes incubated on ice for at least 20 minutes before addition of 3250 µl of chloroform:2-octanol (24:1) solution. The samples were inverted to mix and 250 µL of CTAB/NaCl (10 % CTAB/0.7 M NaCl) solution added. Tubes were incubated at 65 °C for 10 minutes, then centrifuged at 10,000 x g for 5 min at +4 °C. The water phase was transferred to a new tube, mixed with an equal volume of fresh-made phenol:choloroform (1:1) and re-centrifuged. The water phase was transferred to a new tube, mixed with an equal volume of ice-cold chloroform:2-octanol (24:1) and re-centrifuged. The water phase was then mixed with an equal volume of RT isopropanol and incubated at -20 °C for at least 1 h before the DNA sample was collected either by catching it on a glass hook or by centrifugation (10 000 x g, +4 °C, 30 min). The DNA was washed with 70 % ethanol, air dried and suspended in a suitable volume of ultrapure H_2O.

Genome sequencing

Genomic DNA (1 µg) was fragmented in a microTube (100 µL) using Covaris S2 (LGC Genomics). Half of the fragmented DNA (50 µL) was purified using a MinElute Reaction Cleanup kit (Qiagen) and eluted in 25 µL EB buffer. End repair and A-tailing was done on the purified DNA (25 µL) using DNA T4 Polymerase (7.5 U), T4 Polynucleotide Kinase (25 U), dNTP (0.2 mM), DreamTaq DNA Polymerase (1.25 U), ATP (5 mM), 2.5 µL T4 Polynucleotide Kinase Buffer A (10x) and 5 µL T4 DNA Polymerase Buffer, in a total volume of 50 µL (all enzymes from Fermentas). The reaction was incubated for 20 min at 25 ^{0}C, 20 min at 72 ^{0}C and 10 min at 4 ^{0}C. The reaction was

purified using AMPure XP (Beckman Coulter Inc.) and eluted in 32 μL water. Y-Adapters (2 μL 20 μM) were ligated to the purified end-repaired DNA using 4 μL T4 DNA Ligase buffer (10x), 2 μL T4 DNA Ligase (30 U/μL) in a total volume of 40 μL (enzyme from Fermentas) and incubation for 1 h at 25 ^{0}C. The Y-adapters used for HAMBI 540^{T} were 5'-TTCGTATCGCCTCCCTCGCGCCATCAGAGCACTGTAGT -3' and 5'- Phosphate-CTACAGTGCTCTGAGCGGGCTGGCAAGGCGCATAG -3', and for HAMBI 1141 5'-TTCGTATCGCCTCCCTCGCGCCATCAGATCAGACACGT -3' and 5'- Phosphate-CGTGTCTGATCTGAGCGGGCTGGCAAGGCGCATAG -3'. The ligation reaction was purified using AMPure XP and eluted in 25 μL water. A final PCR was done in a 200 μL reaction using DreamTag DNA Polymerase (5 U), 8 pmol A- and B-adapter primers (A, 5'CGTATCGCCTCCCTCGCGCCAT; B, 5'CTATGCGCCTTGCCAGCCCGCT) and 25 μL purified ligation reaction. The PCR cycle was 95 ^{0}C for 3 min, 6 cycles of 95 ^{0}C for 30 s, 60 ^{0}C for 30 s and 72 ^{0}C for 1 min, and final extension at 72 ^{0}C for 5 min. The PCR reaction was purified using AMPure XP and eluted in a volume of 20 μL. The library was checked using Bioanalyzer on a DNA High Sensitive chip (Agilent Technologies). The concentration was measured using a High Sensitive kit on Qubit (Invitrogen).

The 454 sequencing was done according to the manufactures instructions using an Amplicon Lib-A emPCR kit and Titanium Sequencing kit (Roche). SOLiD mate-pair libraries were constructed using a Mate-Paired Library Preparation kit (Life Technologies). From 10 μg of starting material sheared using Covaris S2, libraries were made with insert size 1.5 – 3.5 kb. The obtained libraries were run (50 bp + 50 bp) on a SOLiD 4 DNA Sequencer (Life Technologies). The PacBio libraries (8-10 kb, sheared using g-TUBE, LGC Genomics) were constructed using Library kit from Pacific Biosciences. Two SMRT cells from each strain were run on a PacBio RS instrument (Pacific Biosciences).

The obtained 454 sequences were assembled using Newbler (Roche) yielding a coverage of 17x for HAMBI 1141 and 22x for HAMBI 540^T. The SOLiD mate-pair reads were used to correct the 454 contigs in homopolymer regions and to order the contigs into scaffolds. PCR primers were designed to contig ends and successful PCR products were sequenced using BigDye Chemistry V 3.1 and analysed on an ABI 3130xl capillary sequencer (Life Technologies). Gap4 in the Staden Package [1] was used for editing and manual closing of contig gaps. Gaps that we were not able to close with PCR were closed using the long reads from PacBio which spanned the whole gap. To verify the correctness of the assembly all PacBio reads were mapped to the assembled sequence.

Analysis of evolutionary history of RepABC

Evolutionary analysis of concatenated RepABC proteins of replicons with this replication system in *N. galegae* strains HAMBI 540^T and HAMBI 1141, together with 12 strains representing *Rhizobium*, *Sinorhizobium*, *Mesorhizobium* and *Agrobacterium*, was conducted in MEGA [2]. Accession numbers of the sequences used are listed in Table S3. The evolutionary history was inferred by using the Maximum Likelihood method based on the Jones et al. w/freq. model [3]. The tree with the highest log likelihood (-32712.5611) is shown in Figure S5. The percentage of trees in which the associated taxa clustered together based on bootstrap with 1000 replicates is shown next to the branches. A discrete Gamma distribution was used to model evolutionary rate differences among sites (5 categories (+G parameter = 1.3269)). The tree is drawn to scale, with branch lengths measured in the number of substitutions per site. All sequence positions containing gaps and missing data were eliminated. There were a total of 871 positions in the final dataset.

Analyses of substitution rates and positive selection

The codeml program included in the PAML 4 package [4] was used for analysis of synonymous and non-synonymous substitutions in the protein-coding sequences of the genes shared by HAMBI 540^T and HAMBI 1141 in the symbiosis gene region. The ratio of non-synonymous to synonymous

substitution rates (d_N/d_S or ω) was determined as ML estimation of pairwise comparisons, where one ω was estimated over the whole gene. Sites with ambiguity characters were removed.

For the putative *nifQ* gene, the analysis was expanded to a phylogeny of 10 α-proteobacterial strains (*Azospirillum brasilense* Sp245 [HE577327.1:1280546..1281172] as outgroup, *R. galegae* HAMBI 540^T, *R. galegae* HAMBI 1141, *Rhizobium etli* CFN 42 [U80928.5:279761..280471], *Sinorhizobium fredii* NGR234 [U00090.2:148068..148760], *S. fredii* HH103 [CP003565.1:128216..128935], *Rhizobium tropici* CIAT 899 [CP004017.1:265351..266007], *Mesorhizobium loti* MAFF303099 [NC_002678.2: 4741411..4742058], *Rhizobium mesoamericanum* STM3625 [CANI01000088.1:30510..31142] and *Rhizobium grahamii* CCGE 502 [AEYE02000038.1:26425..27114]) in order to investigate whether positive selection influenced the divergence of *R. galegae nifQ* from the homologs of other rhizobia. The ML phylogenetic tree used as the base for the analyses was constructed from NifQ protein sequences using PhyML [5] at www.phylogeny.fr (19.9.2013), with default options. The unrooted tree without branch lengths was used for codeml analyses. The *nifQ* sequences were analysed for variation in the ratio of non-synonymous to synonymous substitution rates among branches in the NifQ tree, to investigate whether there has been positive selection affecting the evolution of *nifQ* in *R. galegae*. After deletion of alignment gaps, 501 sites were included in the analysis. The null hypothesis (H_0) was that ω is identical across all branches of the *nifQ* phylogeny, i.e. the changes in *R. galegae nifQ* are simply due to an underlying increase in the mutation rate. This null hypothesis was compared to two alternative hypotheses: H_1: ω is variable, being greater than 1 in the branch leading to the two *R. galegae* strains, i.e. there has been a burst of positive selection for functional divergence in that branch and H_2: there has been increased nonsynonymous substitution in both *R. galegae* lineages following divergence from *S. fredii*; hence the assumption of one ω for all branches leading to *R. galegae* and another for the other species. The positive

selection hypothesis was tested against the null hypothesis of neutral evolution through a likelihood ratio test (LRT). Twice the log likelihood difference was compared with χ^2 with d.f. = 1.

noeT mutant construction

The Δ*noeT*::Ω-Km mutant (strain HAMBI 3275) was constructed by marker exchange where the *noeT* gene was replaced with the Ω-Km interposon [6] containing the *nptII* gene. A 1089-bp upstream flanking region of *noeT* (the *noeT* left arm) was amplified with primers HsnTLRBamHI and HsnTLLSpeI-418 and a 1142-bp downstream flanking fragment (the *noeT* right arm) was amplified with primers HsnTRLBamHI and HsnTRRXhoI-4553, using Phusion polymerase (Thermo Scientific). These fragments contained short regions of the 5' and 3' ends respectively, of the gene. The primers contained restriction endonuclease sites (BamHI and SpeI for the left arm and BamHI and XhoI for the right arm) to facilitate directional cloning. The Ω-Km interposon was released from pHP45Ω-Km [6] by BamHI digestion, purified and ligated along with the PCR products (digested with BamHI + SpeI and BamHI + XhoI respectively and purified) into pJQ200SK that had been digested with SpeI and XhoI and dephosphorylated. The resulting construct (pRg103) where the Ω-Km interposon was inserted between the two PCR products was transferred into *Escherichia coli* S17-1 λpir by electroporation (ca 20 ng of pRg103 into 40 µL of electrocompetent cells, electroporation at 2.5 kV, 25 µF and 200 Ω in 0.2 cm spaced cuvettes) and confirmed by sequencing (sequencing primers T3and hsnTL-655 for the left arm; M13 UP and hsnTR-488 for the right arm). The verified construct was then transferred from *E. coli* S17-1 λpir into *R. galegae* HAMBI 1174 by biparental spot mating. Mating was conducted by mixing 1 mL of stationary-phase recipient with 1 mL of donor, pelleting the cells, followed by resuspension in 50 µl of MilliQ water and spotting on a plain TY plate. Exconjugants were plated onto TY agar containing 5 % sucrose and neomycin (25 µg/mL), to select for cells in which the suicide plasmid had been inserted and pJQ200SK removed via recombination events. Mutant candidate clones were colony purified and tested for sensitivity to gentamicin. The final neomycin resistant, gentamicin

sensitive gene replacement mutant, designated HAMBI 3275, was further confirmed by PCR analysis and sequencing. The insert-flanking regions were amplified with three different sets of primers: hsnTmutLL and hsnTmutLR amplifying from within the *Rhizobium* DNA upstream of the left arm to the 5' end of the interposon; primers hsnTmutRL and hsnTmutRR amplifying a fragment from within the 3' end of the interposon to the *Rhizobium* DNA downstream of the right arm; primers hsnTcomF and hsnTcomR amplifying from within the left arm through the Ω-Km fragment into the right arm. These PCR fragments were sequenced over the junctions to confirm that homologous recombination had worked properly. PCR primers used in this work are listed in Table S4.

Nod factor analysis

For extraction of Nod factors from the *R. galegae* HAMBI 1174 *noeT* mutant (HAMBI 3275) and the wild-type parental HAMBI 1174 strain cultures, the strains were grown in 3.15 litres (in aliquots of 350 mL) of def8 medium [7] supplied with neomycin (25 µg/mL), streptomycin (500 µg/mL) and apigenin at a final concentration of 1 µM as inducer of *nod* gene expression. The cultures were incubated at +28 °C on a rotary shaker at 120 rpm. When the cultures reached an OD_{600} value of 1, the cells were spun down at 6000 x g for 15 min at +4 °C, the supernatants were mixed with 0.3 volumes of 1-butanol (Sigma-Aldrich) and stirred for 2 to 3 h. The phases were then allowed to separate overnight before the 1-butanol phase was collected and the samples dried using a rotary evaporator (Büchi Rotavapor R-200 with a vacuum controller V-800).

The crude Nod factor extracts were resuspended in 20 mL of 60 % (v/v) acetonitrile (ACN) (aq) and shaken overnight. Then 10 mL of this solution was diluted with water to a final concentration of 20 % (v/v) ACN (aq). The solution was pre-fractionated using solid phase extraction (SPE) with a C18 cartridge (strata C18-E 70 Å, 5 mL). The Nod factors were eluted from the SPE cartridge with two different ACN (aq) solutions, 45 % and 60 % (v/v), to produce two fractions (5 mL). These SPE fractions were dried down under vacuum, then reconstituted in 700 µL

of 60 % (v/v) ACN (aq) overnight. Prior to reversed-phase high performance liquid chromatography (RP-HPLC), the reconstituted SPE fractions were diluted with water to a final volume of 2 mL. HPLC fractionation was carried out on a C_{18} column (Waters Spherisorb 5μm ODS2, 4.6 x 250 mm) with two subsequent 1 mL injections, eluted using a gradient (20 % ACN (aq) for 20 min, a gradient of 20 % to 60 % ACN (aq) over 20 min) followed by 90 % ACN (aq) for 5 min and 20 % ACN (aq) for 5 min to allow for column re-equilibration. HPLC fractions were collected every minute from the column, eluted at 1 mL min^{-1}. The HPLC fractions corresponding to peaks in UV absorbance of 203 nm were dried under vacuum. The fractions were reconstituted in 50 μL of ACN:H_2O (1:1, v/v) prior to mass spectrometric analysis.

Electrospray ionisation (ESI) mass spectrometric analysis was performed on an Applied Biosystems QSTAR hybrid quadrupole time of flight (QqTOF) tandem mass spectrometer equipped with an ESI source. Analysis was carried out in the positive ion mode. TOF mass spectra were recorded over the range *m/z* 100-1500 and tandem mass spectra were recorded over the range *m/z* 100-1500 with the 'collision offset' set at 80 V. Data were recorded using the Analyst software (version 1.1). Matrix-assisted laser desorption/ionisation (MALDI) QqTOF mass spectrometric analysis was performed on an Applied Biosystems QSTAR hybrid QqTOF tandem mass spectrometer equipped with an o-MALDI source. Analysis was carried out in the positive ion mode. TOF mass spectra were recorded over the range *m/z* 600-3000 and tandem mass spectra were recorded over the range *m/z* 200-3000 with the 'collision offset' set at 80 V. The sample spots on the MALDI plate were irradiated with a nitrogen UV laser (337 nm wavelength) with a pulse rate of 20 Hz. The power level was 100 μJ. Data were recorded with the Analyst software (version 1.1).

Aliquots (20 μL) of the HPLC fractions containing *O*-acetylated nod factors were dried under vacuum. Methanol (250 μL) and concentrated ammonia (35 % v/v, 250 μL) were added to each aliquot, mixed, and were left overnight. The fractions were dried under vacuum and then reconstituted in 50 μL of ACN:H_2O (1:1, v/v) prior to MALDI mass spectrometric analysis.

Plant tests of the *noeT* mutant

For nodulation tests, seeds of *G. orientalis, Trifolium repens* cv. Milka, *Pisum sativum* cv. Afghanistan (provided by Mike Ambrose, John Innes Centre), *Phaseolus vulgaris* var. Nanus, *Vicia hirsuta* and *A. sinicus* were surface sterilised by washing the seeds in 96 % ethanol for 1 minute, 3 % sodium hypochlorite for 3-5 minutes and washing with sterile water 5-6 times for 1-2 minutes. The sterilised seeds were germinated on TY or YEM agar plates at room temperature in darkness. *G. officinalis* seeds were surface sterilised by the following procedure: washed with concentrated sulphuric acid for 15 minutes, rinsed with sterile water 8 times for 2 minutes, kept in 96 % ethanol for 1 minute and finally washed with sterile water 6 times for 2-5 minutes.

In order to compare the nodulation phenotype of the *noeT* mutant to the wild-type parental strain, *G. orientalis* seeds were transferred into separate test tubes (2 cm diameter, 15 cm high) containing 10-12 mL of quarter-strength nitrogen-free Jensen medium [8] and inoculated with 20 flL of late log-phase HAMBI 1174 or mutant culture. Initially 20 seedlings were prepared of each kind, but one mutant-inoculated plant was later discarded due to clearly abnormal growth. Prior to inoculation the bacterial cultures were re-suspended in sterile MilliQ water. Cultures in which antibiotics had been used were washed once in an equal volume of sterile MilliQ water before final re-suspension. Negative control seedlings were inoculated with 20 flL of sterile MilliQ water. Nodule formation was recorded from tubes daily during the first 23 days post inoculation (dpi), then sporadically until 40 dpi. IBM SPSS Statistics for Windows version 20.0 (Armonk, NY: IBM Corp.) was used for analyses of statistical significance.

Conjugation experiments

Strains used to test the conjugative properties of *N. galegae* sv. officinalis strain HAMBI 1141 are listed in Table S1. Culture media were supplemented with appropriate antibiotics at the following concentrations: ampicillin (Ap) 100 µg/mL, gentamicin (Gm) 25 µg/mL, kanamycin (Km) 50 µg/mL, neomycin (Nm) 25 or 50 µg/mL, rifampicin (Rf) 150 µg/mL, spectinomycin (Spc) 500

µg/mL, streptomycin (Sm) 1000 µg/mL, tetracycline (Tc) 10 µg/mL. The GusA substrate X-Gluc (5-bromo-4-chloro-3-indoxyl-β-D-glucuronidase) was supplied at 50 µg/mL. Media of *E. coli* ST18 were supplemented with 50 µg/mL of 5-aminolevulinic acid (ALA).

Biparental mating of HAMBI 1207 and HAMBI 1587 was done by growing the cultures to an OD_{600} of about 1, then mixing 1mL of HAMBI 1207 culture with 1 mL of HAMBI 1587 and resuspending the combined pellet in 100 flL H_2O. Spot mating was performed by adding the resuspended cells onto a TY plate, and incubating at +28 °C for 48 h. The spot was then resuspended in 1 mL H_2O and dilutions 10^{-6} to 10^{-9} were made. A 100 flL aliquot of each dilution was spread onto TY+Nm plates and incubated at +28 °C for 3 days. The remaining 990 flL of the undiluted mating spot suspension was centrifuged and resuspended in 100 flL H_2O, spread onto a TY+Nm plate and incubated at +28 °C for 3 days (hereafter called ”undiluted” plate). Plates 10^{-6} (385 colonies) and the ”undiluted” plate were used for inoculation of plants. There were 5 replicates for each type to test: undiluted exconjugants on *G. orientalis*, undiluted exconjugants on *G. officinalis*, 10^{-6} diluted exconjugants on *G. orientalis* and *G. officinalis*. One negative control was prepared for each test type. *G. orientalis* and *G. officinalis* seeds were sterilised and germinated as described in section ”Plant tests of the *noeT* mutant”. All colonies on the 10^{-6} and ”undiluted” plates were suspended in H_2O. The OD_{600} of the ”undiluted” suspension was 2.4. Single plants in glass tubes (sterilized glass test tubes with each tube containing one seedling on a metal mesh covered with about 10-12 mL of quarter-strength nitrogen-free Jensen nutrient solution [8]) were inoculated with 20 flL per plant of either suspension, or sterile water for the controls. Inoculated plants were maintained in a growth chamber (20°C for 1 h, 24 °C for 16 h, 20 °C for 1h and 16°C for 6h). After 3 to 4 weeks, nodules were isolated from plants, sterilised (using the same procedure as for *G. orientalis* seeds) and crushed in 100 flL H_2O. From this, 50 flL was streaked onto both a plein TY plate and a TY+Sm+Nm plate, and incubated at +28 °C for 3 days. From the colonies that arose on the selective plates, single colonies were used to inoculate TY broths for re-inoculation on the same

plants to check that nodulation occurs again. The OD_{600} values of cultures used for re-inoculation was 0.8 to 1.0. Again, 20 flL of each culture was used to inoculate single plants in tubes.

After 6 weeks, nodules were observed only on those plants inoculated with ”undiluted” samples. Nodules were isolated, sterilised and bacteria isolated and spread onto selective plates using the same procedure as previously. Arising colonies were purified on new selective plates, and 5 transconjugant clones (two isolated from *G. orientalis* and 3 isolated from *G. officinalis*) were retained for further experiments. These transconjugant clones were tested once more on both *G. orientalis* and *G. officinalis,* in jar experiments. Jars were prepared by filling them with leca gravel (4-10 mm), sand (0.5-1.2 mm) and vermiculite (1-2 mm) mixed at a ratio of 3:5:5. Jars were then sterilised at 160 °C for 24 h. Germinated seeds were transferred to jars aseptically, 3 seeds per jar. Jars had been moistened beforehand with 125 mL of quarter-strength nitrogen-free Jensen nutrient solution, and 50 mL H_2O was added after covering the seeds. The plants were provided with 30-40 mL of quarter-strength Jensen nutrient solution twice a week. Control jars of HAMBI 1587 on both *G. orientalis* and *G. officinalis* were also prepared. All plants were grown for 4 weeks before roots were examined for nodules. A modified Eckhardt gel [9] was run to confirm the plasmid profile of the 5 transconjugants. The transconjugant strains as well as their reference strains were grown overnight in HP medium [16] to an OD_{600} of approximately 0.3. Bacterial cultures of 250 µL for each strain were added to 500 µL of a 0.3 % (w/v) Sarkosyl solution. Each sample was mixed, pelleted by centrifugation for 4 min and resuspended in 25 µL of lysis solution (10 % (w/v) sucrose, 100 µg/mL lysozyme, 10 µg/mL RNase in Tris-EDTA (TE) or 1 x TBE buffer). The resuspensions were mixed with 5 µL of 6 × DNA Loading Dye and immediately loaded onto a 0.7 % agarose gel containing 1 % (w/v) SDS in 1 × TBE buffer. Electrophoresis was performed at 60 V (ca. 20 mA) at 4 °C for 16-18 h. The gel was stained for 30 min in a 0.1 µg/mL ethidium bromide solution prior to imaging. This analysis confirmed that each of the isolates had obtained the smaller plasmid from strain HAMBI 1207.

Similar biparental matings were performed between HAMBI 1207 and HAMBI 1218, HAMBI 1141 and HAMBI 1213, HAMBI 1141 and HAMBI 1594 to study the transfer of the symbiosis plasmid to other rhizobial species. Exconjugants of these matings were all inoculated on *G. officinalis* but also on *M. sativa* when HAMBI 1218 or HAMBI 1213 was the donor, or on *V. villosa* when HAMBI 1594 was the donor. *M. sativa* and *V. villosa* seeds were sterilized using the same method as used for *G. orientalis*, with an additional incubation in water for 4 h for *V. villosa* prior to transfer of the seeds to TY plates. Inoculations were done by applying 20 flL of resuspended cells onto single plants in glass tubes.

A deletion mutant was constructed in HAMBI 1141 to study the impact of the T4SS located on the chromid, on conjugation of the symbiosis plasmid. To incorporate *lox* sites flanking the HAMBI1141 deletion target, the T4SS on the chromid, an upstream sequence of 1331 bp and a downstream sequence of 1658 bp were amplified using designed primers pairs (ZZ01-ZZ08 for the upstream fragment and ZZ03-ZZ04 for the downstream fragment) having a BamHI restriction site incorporated in the forward primers and a NotI site in the reverse primers (Table S4). PCR was done using Phusion Polymerase (Thermo Scientific), with ca. 40 ng of genomic DNA of HAMBI 1141 used as template. The obtained PCR products were purified and cloned into a pJET1.2/blunt vector using the Clone JET Cloning kit (Fermentas) according to the manufacturer's instructions. The inserted products were verified by sequencing using primers provided in the kit. The verified upstream and downstream PCR products were both digested with FastDigest BamHI and FastDigest NotI (Fermentas) restriction endonucleases. The digests were purified and confirmed by agarose gel electrophoresis. Plasmids pAL01_MCS1 and pAL02/2_MCS2 carrying the *lox71* and *lox66* site respectively, were digested with BamHI and NotI restriction enzymes in sequential reactions, dephosphorylated and purified. The prepared upstream and downstream PCR inserts (ca. 20 ng) were ligated into digested vector pAL01_MCS1 and pAL02/2_MCS2 (ca. 20 ng) respectively, resulting in two new vectors named pZZlox71 and pZZlox66. The ligations were precipitated and

dissolved in 5 µL of water. The constructed pZZlox71 and pZZlox66 plasmids were transformed into electrocompetent *E. coli* ST18 cells by electroporation (electroporation at 2.5 kV, 25 µF and 250 Ω). The transformed cells were incubated at 37 ˚C for 1 h and plated on selective media. Transformants were verified by plasmid profile analysis of BamHI and NotI single and double digestions of isolated plasmids. To integrate plasmids pZZlox66 and pZZlox71 into the chromid of HAMBI 1141, verified *E. coli* ST18 clones containing pZZlox66 or pZZlox71 as donors were mated with HAMBI 1141 as the recipient sequentially. First, 400 µL of washed *E. coli* ST18(pZZlox66) donor strain culture (OD_{600} 0.35) was mixed with 1 mL of the recipient strain HAMBI 1141 (OD_{600} 1.2). The pelleted cells were suspended in 50 µL of sterile water, spotted on a TY+ALA plate and incubated at 28 °C overnight. The cells were collected from the mating spot and resuspended in 1 mL of water. Following dilution, the cells were spread onto selective plates (TY+Gm) and incubated for 3 days. The integration of pZZlox66 into the chromid was verified by PCR with primers AL064 and ZZ10 (Table S4). After pZZlox66 integration had been confirmed by PCR, the HAMBI 1141 single plasmid insertion mutant and the *E. coli* ST18(pZZlox71) donor were grown to stationary phase and mixed at a 1:5 ratio of donor to recipient. The mixed cells were treated as described above and transconjugants were selected on TY+Nm+Gm plates containing GusA substrate. A blue colony that arose on a selective plate after three days was picked and its genomic DNA was isolated, followed by PCR amplification of a 1.7-kb fragment consisting of genetic regions from both pZZlox71 and the chromid (primers ZZ09 and AL062, Table S4). With the same genomic template, PCR verification of the first integration was performed again to ensure that both plasmids have integrated into the chromid of the same strain. The PCR products were then sequenced with primer pairs AL064 and ZZ10 for the first integration and ZZ09 with a universal primer RP-48 for the second integration (Table S4). The verified HAMBI 1141 co-integrate strain was then mated with *E. coli* ST18 carrying pCM157(p*nirS*) at a ratio of 5:2. Transconjugants appearing on selective plates were tested for excision of the targeted region by verifying loss of

their Nm and Gm resistance. Precise excision was validated by PCR amplification of a 1.3 kb fragment spanning part of both the upstream and downstream regions with primer pair ZZ11 and ZZ12. The resulting PCR product was sequenced using primer ZZ13 (Table S4). Finally, the deletion mutant strain was cultivated on plain TY plates to eliminate pCM157(p*nirS*).

HAMBI 1207 was cured of its symbiosis plasmid in order to study the ability of this strain to regain its symbiosis functions through conjugation with corresponding wild-type and mutant strains. Transposon Tn*5*-B12S carried on pMH1701 was used to randomly label the replicons of HAMBI 1207 through mating between an *E. coli* strain that harbours pMH1701 and HAMBI 1207. Log-phase donor and recipient were mixed at a ratio of 1:5. HAMBI 1207 transposon insertion mutants were selected on TY+Nm+Sm plates and colonies were picked at random after three days of incubation. After purification, triparental matings were carried out between the donor strain HAMBI 1207 insertion mutants, the plasmid free recipient strain *Agrobacterium fabrum* UBAPF2 and a helper strain *E.coli* J-53(RP4-4) at a ratio of 2:2:1. *Agrobacterium* transconjugants were identified by selection on TY+Nm+Rf plates. After colony purification, *Agrobacterium* transconjugants were run on a modified Eckhardt gel (described above) to detect the presence of plasmids. HAMBI 1207 insertion mutants which were identified as carrying the transposon insert on the symbiosis plasmid were plated on TY plates containing 5 % sucrose, after appropriate dilutions. These mutants were first incubated at 37 °C for two days and then at 28 °C for a further three days. Colonies found on sucrose-containing plates accompanied by the loss of neomycin resistance were picked and subjected to a modified Eckhardt gel electrophoresis (described above) to further confirm the change of their plasmid pattern.

Biparental matings of the plasmid-cured HAMBI 1207 (HAMBI 3489) and the HAMBI 1141 strain where the T4SS gene region on the chromid was deleted (HAMBI 3490) were performed by mixing 1 mL each of stationary-phase bacterial cultures and preparation of a mating spot as described above. A corresponding mating was done between wild-type HAMBI 1141 donor

and HAMBI 3489 recipient strain. Cells that had been diluted 10^{-4} were flushed from the surface of the selective plates with TY broth. The suspensions were diluted to an OD_{600} of about 1.8 prior to inoculation on *G. officinalis* plants in a jar set-up as described above. Three seeds in a jar were inoculated with 1 mL of mated bacterial cells. *G. officinalis* seedlings were also inoculated with 50 µL of bacterial cells and grown in glass tubes, 3 tubes per mating type. Sterile water was used to inoculate negative control plants. Inoculated plants in both jars and test tubes were maintained in a growth chamber as described above. Examination of nodulation was carried out 2 weeks post inoculation for plants grown in glass tubes and 4 weeks post inoculation for those grown in jars, by visually inspecting the roots of plants.

Gentamicin resistance was introduced to HAMBI 3489 by introducing pZZlox66 into the chromid (biparental mating of cultures with an OD_{600} about 0.9, mixed at a ratio 1:1). Colonies that appeared on selective plates were purified and tested on def8 and LB+ALA media. Clones were verified by PCR verification from genomic DNA. This strain, HAMBI 3491, was used for biparental matings with HAMBI 1141, HAMBI 3470 and HAMBI 3490 as the donors. Biparental spot matings of stationary-phase cultures were performed mixed 1:1 as described above. Biparental mating of HAMBI 3490 and HAMBI 1587 was also done in the corresponding way. Cells from a 10^{-5} dilution plate as well as an ”undiluted” plate of each mating (as described for the mating of HAMBI 1207 and HAMBI 1587 above) were used to inoculate *G.officinalis* plants (*G.orientalis* for the HAMBI 3490-HAMBI 1587 exconjugants). The OD_{600} values for the suspensions used were 1.4-1.7. These suspensions were washed once with water before final resuspension in 1 mL H_2O. Strains HAMBI 3491, HAMBI 3490, HAMBI 1141 and the HAMBI 1141 pZZlox71-pZZlox66 co-integrate strain were used as control inoculants. Again 20 µL of each sample was used to inoculate plants grown in tubes. Where nodules were formed, nodules were isolated, sterilised and crushed in 50 µL H_2O. From this suspension, 20 µL was transferred onto a TY or YEM plate and 20 µL onto a TY+Gm plate to select for HAMBI 3491. Colonies that appeared were purified and cultured in HP

broth for plasmid gel analysis as described above (modified Eckhardt gel), with HAMBI 3491 and HAMB 3470 used as reference strains. From the HP broth, 100 f!L was also used to inoculate TY broths to use for re-inoculation of plants.

Table S1. Strains and plasmids used in this study

strain or plasmid	characteristics	reference/source
**strains**		
N. galegae		
HAMBI 540^{T}	sv. orientalis. Type strain of *N. galegae*	[7, 10]
HAMBI 1174	Sm^{r}, Spc^{r} derivative of HAMBI 540^{T}	[11]
HAMBI 1141	sv. officinalis	[7, 10]
HAMBI 1207	Sm^{r} derivative of HAMBI 1141	K. Lindström
HAMBI 3275	HAMBI 1174 Δ*noeT*::Ω-Km mutant. Sm^{r}, Spc^{r}, Nm^{r}	This work
HAMBI 1587	HAMBI 1174::Tn5 in nodABC. Sm^{r}, Spc^{r}, Nm^{r}	[12]
HAMBI 3489	HAMBI 1207 cured of symbiosis plasmid. Sm^{r}	This work
HAMBI 3470	HAMBI 1587 containing symbiosis plasmid of HAMBI 1207. Nm^{r}	This work
HAMBI 3490	HAMBI 1141 chromid T4SS deletion mutant	This work
HAMBI 3491	HAMBI 3489 containing pZZlox66. Gm^{r}	This work
R. tropici		
HAMBI 1163	Type strain of *R. tropici*. CIAT 899	[13]
R. leguminosarum		
HAMBI 1594	RBL5515 pRL1JI*nodA*10::Tn5. Rif^{r}, Nod^{-}	[14]
A. fabrum		
HAMBI 1218	C58 cured of Ti plasmid	[15]
UBAPF2	Plasmid-free, Rf^{r}	[16]
S. meliloti		
HAMBI 1213	*S. meliloti* 1021::Tn5 in *nodC*. Str^{r}, Nod^{-}	[17]
E. coli		
S17-1 λpir	Tp^{r}, Sm^{r}, *recA, pro, thi, hsdR*$^{-}$ *M*$^{+}$, RP4-2-Tc::Mu-Km::Tn*7*, λ*pir* lysogen	[18] Provided by Clive Ronson lab
ST18	S17 λ-pir ΔhemA. ALA	[19]
**plasmids**		
pJQ200SK	Suicide vector, P15A *oriV sacB mob,* Gm^{r}	[20] Provided by Clive Ronson lab
pHP45Ω-Km	Source of Ω-Km interposon, Km^{r}	[6] Provided by Clive Ronson lab
pRg103	pJQ200SK containing the Ω-Km interposon flanked by upstream and downstream regions of the HAMBI 1174 *noeT* gene.	This work
RP4-4	helper plasmid. Nm^{s}, Ap^{r}, Tc^{r}	[21]
pMH1701	Suicide vector, carries Tn*5*-B12S. Km^{r}	[22]
pCM157(p*nirS*)	*cre* gene under *nirS* promotor of *M. gryphiswaldense*	[23]
pAL01_MCS1	Carries *gusA* gene and *lox 71* site, Km^{r}	[23]
pAL02/2_MCS2	Carries *lox 66* site, Gm^{r}	[23]
pZZlox66	pAL02/2_MCS2 carrying HAMBI 1141 chromid T4SS downstream fragment as BamHI-NotI insert	This work
pZZlox71	pAL01_MCS1 carrying HAMBI 1141	This work

	chromid T4SS upstream fragment as BamHI-NotI insert	

Sm: streptomycin; Spc: spectinomycin; Tp: trimethoprim; Nm: neomycin; Km: kanamycin; Gm: gentamicin; Rf: rifampicin, Ap: ampicillin.

Table S2. Accession numbers of reference genomes used

**Strain**	**BioProject**	**RefSeq**
R. leguminosarum sv. viciae 3841	PRJNA57955	NC_008378-NC_008384
S. meliloti 1021	PRJNA57603	NC_003037, NC_003047, NC_003078
A. fabrum C58	PRJNA57865	NC_003062-NC_003065
R. tropici CIAT 899	PRJNA185179	NC_020059-NC_020062
S. medicae WSM419	PRJNA58549	NC_009636, NC_009620-NC_009622
R. leguminosarum sv. trifolii WSM2304	PRJNA58997	NC_011366, NC_011368-NC_011371
R. phaseoli CIAT 652	PRJNA59115	NC_010994, NC_010996-NC_010998
R. etli sv. mimosae str. Mim1	PRJNA213896	NC_021905-NC_021911
R. etli CFN 42	PRJNA58377	NC_007761-NC_007766, NC_004041.2
S. fredii NGR234	PRJNA59081	NC_012587, NC_012586, NC_000914.2
M. ciceri sv. biserrulae WSM1271	PRJNA62101	NC_014923, NC_014918
R. rhizogenes K84	PRJNA58269	NC_011983, NC_011985, NC_011987, NC_011990, NC_011994

Table S3. Accession numbers of RepABC sequences used

Strain	replicon	operon	accession numbers
R. leguminosarum sv. viciae 3841	pRL7	RepABC	YP_770746-YP_770748
	pRL8	RepABC	YP_770902-YP_770904
	pRL9	RepABC	YP_765299-YP_765301
	pRL10	RepABC	YP_770304-YP_770306
	pRL11	RepABC	YP_771034-YP_771036
	pRL12	RepABC	YP_764518-YP_764520
R. leguminosarum sv. trifolii WSM2304	pRLG201	RepABC	ACI58641-ACI58643
	pRLG202	RepABC	YP_002278285-YP_002278287
	pRLG203	RepABC	YP_002283933-YP_002283935
	pRLG204	RepABC	YP_002284251-YP_002284253
R. tropici CIAT 899	pRtrCIAT899a	RepABC	YP_007335859-YP_007335861
	pRtrCIAT899b	RepABC	YP_007336359-YP_007336361
	pRtrCIAT899c	RepABC	AGB75718-AGB75720
R. phaseoli CIAT 652	pA	RepABC	YP_001985988-YP_001985990
	pB	RepABC	YP_001984631-YP_001984633
	pC	RepABC1	ACE94483-ACE94485
		RepABC2	ACE95057-ACE95059
R. etli sv. mimosae str. Mim1	pRetMIM1a	RepABC	YP_008367050-YP_008367052
	pRetMIM1b	RepABC	YP_008367283-YP_008367285
	pRetMIM1c	RepABC	YP_008368599-YP_008368601
	pRetMIM1d	RepABC	YP_008367757-YP_008367759
	pRetMIM1e	RepABC	YP_008368337-YP_008368339
	pRetMIM1f	RepABC1	AGS26678-AGS26680
		RepABC2	AGS26377-AGS26379
R. etli CFN 42	p42a	RepABC1	YP_471769-YP_471771
		RepABC2	YP_471629-YP_471631
	p42b	RepABC	YP_471932-YP_471934
	p42c	RepABC	YP_472164-YP_472166
	p42d	RepABC	NP_660040-NP_660042
	p42e	RepABC	YP_472619-YP_472621
	p42f	RepABC1	YP_473186-YP_473188
		RepABC2	YP_472830-YP_472832
S. fredii NGR234	pNGR234a	RepABC	NP_443801-NP_443803
	pNGR234b	RepABC	ACP21477-ACP21479
S. meliloti 1021	pSymA	RepABC	AAK65950-AAK65952
	pSymB	RepABC	CAC48447-CAC48449
S. medicae WSM419	pSMED01	RepABC	ABR62896-ABR62898
	pSMED02	RepABC	ABR63950-ABR63952
	pSMED03	RepABC	YP_001314978-YP_001314980
M. ciceri sv. biserrulae WSM1271	pMESCI01	RepABC	YP_004134557-YP_004134559
R. rhizogenes K84	pAtK84b	RepABC	YP_002551334-YP_002551336
	pAtK84c	RepABC	YP_002546569-YP_002546571
A. fabrum C58	pC58Ti	RepABC	NP_396560-NP_396562

	pC58At	RepABC	NP_395939-NP_395941

Table S4. Primers used in this study

**primer**	**sequence 5’-3’**	**reference**
HsnTLRBamHI	AAATTTGGATCCGCGGCATAGAAGACCTGATG	
HsnTLLSpeI-418	TTTAAAACTAGTCCTCCTTGCAGTCATAGATC	
HsnTRLBamHI	AAATTTGGATCCGCTCGTTAGAGCATTTTCGA	
HsnTRRXhoI-4553	TTTAAACTCGAGCTCCAGTCAATTCCGGACAG	
hsnTL-655	CTGTTCATTTGCACTTAGAGC	
hsnTR-488	ATAACTCTCAGCCACAATCG	
hsnTmutLL	GCATTATCTGGTCGTGTATG	
hsnTmutLR	ACTATCAGGTCAAGTCTGCT	
hsnTmutRL	TTGATGTTACCCGAGAGCTT	
hsnTmutRR	ACGACATGCTGTCATAGAAG	
hsnTcomF	AAATTTAAGCTTCATTTGCACTTAGAGCTCAA	
hsnTcomR	TTTAAAAAGCTTTTGAAACAGAGTCTATCTCG	
ZZ01	TTTAAAGGATCCGGTTCTCGGTCAGGTTGGAG	
ZZ08	AAATTTGCGGCCGCACCGGATTCGAGGTTCGCTC	
ZZ03	TTTAAAGGATCCGATGATGGCAAGTTCGGCAC	
ZZ04	AAATTTGCGGCCGCCAATCACCAATGCGCTCCTG	
AL064	CAGATTACGGTGACGATCCC	[23]
ZZ10	CGTGGAGATTTCGGCCTATG	
ZZ09	CAGCCATCGCGTAACGTTG	
AL062	CGCAACGCAATTAATGTGAG	[23]
RP-48	AGCGGATAACAATTTCACACAGGA	
ZZ11	CGATCGGTGTAGAAGGTCTG	
ZZ12	AGTGAGAAGTCCAGCTTCAG	
ZZ13	CCGAAGCACGTGACATCCTG	

References

1. Staden R, Beal KF, Bonfield JK: **The Staden package, 1998**. In *Methods in Molecular Biology*. Volume 132. Edited by Misener S, Krawets SA. Totowa, New Jersey: Humana Press; 2000:115-130.

2. Tamura K, Peterson D, Peterson N, Stecher G, Nei M, Kumar S: **MEGA5: Molecular Evolutionary Genetics Analysis using Maximum Likelihood, Evolutionary Distance, and Maximum Parsimony Methods**. *Mol Biol Evol* 2011, **28:**2731-2739.

3. Jones DT, Taylor WR, Thornton JM: **The rapid generation of mutation data matrices from protein sequences**. *Comput Appl Biosci* 1992, **8:**275-282.

4. Yang Z: **PAML 4: Phylogenetic Analysis by Maximum Likelihood.** *Mol Biol Evol* 2007, **24:**1586-1591.

5. Guindon S, Dufayard J, Lefort V, Anisimova M, Hordijk W, Gascuel O: **New algorithms and methods to estimate maximum-likelihood phylogenies: assessing the performance of PhyML 3.0.** *Syst Biol* 2010, **59:**307-321.

6. Fellay R, Frey J, Krisch H: **Interposon mutagenesis of soil and water bacteria: a family of DNA fragments designed for in vitro insertional mutagenesis of Gram-negative bacteria.** *Gene* 1987, **52:**147-154.

7. Lindström K, Lehtomäki S: **Metabolic properties, maximum growth temperature and phage sensitivity of *Rhizobium* sp. (*Galega*) compared with other fast-growing rhizobia.** *FEMS Microbiol Lett* 1988, **50:**277-287.

8. Vincent JM: *A manual for the study of root-nodule bacteria (IBP Handbook No. 15)*. Oxford: Blackwell Scientific Publications; 1970.

9. Eckhardt T: **A rapid method for the identification of plasmid deoxyribonucleic acid in bacteria.** *Plasmid* 1978, **1:**584-588.

10. Radeva G, Jurgens G, Niemi M, Nick G, Suominen L, Lindström K: **Description of two biovars in the *Rhizobium galegae* species: biovar orientalis and biovar officinalis.** *Syst Appl Microbiol* 2001, **24:**192-205.

11. Lindström K, Sarsa M, Polkunen J, Kansanen P: **Symbiotic nitrogen fixation of *Rhizobium* (Galega) in acid soils, and its survival in soil under acid and cold stress.** *Plant Soil* 1985, **87:**293-302.

12. Suominen L, Roos C, Lortet G, Paulin L, Lindström K: **Identification and structure of the *Rhizobium galegae* common nodulation genes: evidence for horizontal gene transfer.** *Mol Biol Evol* 2001, **18:**907-916.

13. Martínez-Romero E, Segovia L, Mercante FM, Franco AA, Graham P, Pardo MA: ***Rhizobium tropici*, a novel species nodulating *Phaseolus vulgaris* L. beans and *Leucaena* sp. trees.** *Int J Syst Bacteriol* 1991, **41:**417-426.

14. Wijffelman CA, Pees E, van Brussel AAN, Okker RJH, Lugtenberg BJJ: **Genetic and functional analysis of the nodulation region of the *Rhizobium leguminosarum* Sym plasmid pRL1JI.** *Arch Microbiol* 1985, **143:**225-232.

15. van Larebeke N, Engler G, Holsters M, van den Elsacker S, Zaenen I, Schilperoort RA, Schell J: **Large plasmid in *Agrobacterium tumefaciens* essential for crown gall-inducing ability.** *Nature* 1974, **252:**169-170.

16. Hynes MF, Simon R, Pühler A: **The development of plasmid-free strains of *Agrobacterium tumefaciens* by using incompatibility with a *Rhizobium meliloti* plasmid to eliminate pAtc58.** *Plasmid* 1985, **13:**99-105.

17. Meade HM, Long SR, Ruvkun GB, Brown SE, Ausubel FM: **Physical and genetic characterization of symbiotic and auxotrophic mutants of *Rhizobium meliloti* induced by transposon Tn5 mutagenesis.** *J Bacteriol* 1982, **149:**114-122.

18. Simon R, Priefer U, Pühler A: **A broad host range mobilization system for in vivo genetic engineering: transposon mutagenesis in Gram negative bacteria.** *Nat Biotech* 1983, **1:**784-791.

19. Thoma S, Schobert M: **An improved *Escherichia coli* donor strain for diparental mating.** *FEMS Microbiol Lett* 2009, **294:**127-132.

20. Quandt J, Hynes MF: **Versatile suicide vectors which allow direct selection for gene replacement in gram-negative bacteria.** *Gene* 1993, **127:**15-21.

21. Simon R: **High frequency mobilization of gram-negative bacterial replicons by the in vitro constructed Tn5-Mob transposon.** *Mol Gen Genet* 1984, **196:**413-420.

22. Hynes MF, Quandt J, O'Connell MP, Pühler A: **Direct selection for curing and deletion of *Rhizobium* plasmids using transposons carrying the *Bacillus subtilis* sacB gene.** *Gene* 1989, **78:**111-120.

23. Lohße A, Ullrich S, Katzmann E, Borg S, Wanner G, Richter M, Voigt B, Schweder T, Schüler D: **Functional analysis of the magnetosome island in *Magnetospirillum gryphiswaldense*: the *mamAB* operon is sufficient for magnetite biomineralization.** *PLoS ONE* 2011, **6:**e25561.
